# Effects of Siberian fir terpenes extract Abisil on antioxidant activity, autophagy, transcriptome and proteome of human fibroblasts

**DOI:** 10.18632/aging.203448

**Published:** 2021-08-24

**Authors:** Anastasiya Lipatova, George Krasnov, Pavel Vorobyov, Pavel Melnikov, Olga Alekseeva, Yulia Vershinina, Alexander Brzhozovskiy, Daria Goliusova, Faniya Maganova, Natalia Zakirova, Anna Kudryavtseva, Alexey Moskalev

**Affiliations:** 1 Engelhardt Institute of Molecular Biology, Russian Academy of Sciences, Moscow 119991, Russia; 2 Center for Precision Genome Editing and Genetic Technologies for Biomedicine, Engelhardt Institute of Molecular Biology, Russian Academy of Sciences, Moscow 119991, Russia; 3 V. Serbsky National Research Center for Psychiatry and Narcology, Moscow 119034, Russia; 4 Skolkovo Institute of Science and Technology, Moscow 121205, Russia; 5 Vavilov Institute of General Genetics, Russian Academy of Sciences, Moscow 119991, Russia; 6 Initium-Pharm, LTD, Moscow 142000, Russia; 7 Institute of Biology of Federal Research Center “Komi Science Center” of Ural Branch of RAS, Syktyvkar 167982, Russia; 8 Russian Clinical and Research Center of Gerontology, Moscow 129226, Russia

**Keywords:** terpenes, autophagy, proteome, fibroblasts, transcriptome

## Abstract

Background: Abisil is an extract of Siberian fir terpenes with antimicrobial and wound healing activities. Previous studies revealed that Abisil has geroprotective, anti-tumorigenic, and anti-angiogenic effects. Abisil decreased the expression of cyclin D1, E1, A2, and increased the phosphorylation rate of AMPK.

Objective: In the present study, we analyzed the effect of Abisil on autophagy, the mitochondrial potential of embryonic human lung fibroblasts. We evaluated its antioxidant activity and analyzed the transcriptomic and proteomic effects of Abisil treatment.

Results: Abisil treatment resulted in activation of autophagy, reversal of rotenone-induced elevation of reactive oxygen species (ROS) levels and several-fold decrease of mitochondrial potential. Lower doses of Abisil (25 μg/ml) showed a better oxidative effect than high doses (50 or 125 μg/ml). Estimation of metabolic changes after treatment with 50 μg/ml has not shown any changes in oxygen consumption rate, but extracellular acidification rate decreased significantly. Abisil treatment (5 and 50 μg/ml) of MRC5-SV40 cells induced a strong transcriptomic shift spanning several thousand genes (predominantly, expression decrease). Among down-regulated genes, we noticed an over-representation of genes involved in cell cycle progression, oxidative phosphorylation, and fatty acid biosynthesis. Additionally, we observed predominant downregulation of genes encoding for kinases. Proteome profiling also revealed that the content of hundreds of proteins is altered after Abisil treatment (mainly, decreased). These proteins were involved in cell cycle regulation, intracellular transport, RNA processing, translation, mitochondrial organization.

Conclusions: Abisil demonstrated antioxidant and autophagy stimulating activity. Treatment with Abisil results in the predominant downregulation of genes involved in the cell cycle and oxidative phosphorylation.

## INTRODUCTION

Life expectancy in economically developed countries gradually increases along with the burden of chronical diseases [1]. However, centenarians have several times higher delay of aging-related diseases [2]. It is well documented that some mammals live not only unusually longer but are also resistant to stress, tumor diseases and type 2 diabetes mellitus [3]. Based on these discoveries, geroscience conception suggests that by focusing not on specific diseases of aging, but on targeting the mechanisms of aging itself, it is possible to achieve more significant effects of prolonging a healthy period of life [4]. It may be promising to identify the effects of pharmacological compounds on gene activity patterns associated with molecular pathways of aging and longevity.

Substances that can slow down aging, the so-called geroprotectors, delay the onset of age-related diseases and reduce the mortality risks. It should be noted that potential geroprotectors could be not only synthetic drugs. Of greatest interest are sources of potential geroprotectors of natural origin, primarily from already known medicinal and food plants. They are the most tolerable and faster to put on a market.

At present, the geroprotective properties are revealed by the increase in the lifespan of model organisms. From this point of view such properties of many plant extracts were studied [5]. Another criteria for anti-aging drug is the effect on the basic molecular and cellular processes of aging [6]. A variety of studies has demonstrated that disruption of cellular homeostasis and molecular stability through increased formation of free radicals, lipid peroxidation, and protein glycoxidation is important for cellular senescence and organismal aging [7] confirming the theory of oxidative stress, proposed by Denham Harman [8], to be one of the mechanisms of aging. Nevertheless, the antioxidant properties of the molecules *in vitro* are unlikely to explain most beneficial pharmacological effects *in vivo* [9]. It is more probable that such compounds as xenobiotics trigger a mild oxidative stress and then the expression of genes of internal antioxidant defense, mitohormesis [10] or other stress resistance pathways, such as autophagy [11] or positive epigenetic shift [12]. A decrease in the activity of the electron transport chain and removal of defective mitochondria are the key modulators of oxidative stress reaction [13]. Inducible mitophagy can play a decisive role in reducing the formation of free radicals [14, 15].

Our previous studies have shown that Abisil, an extract of Siberian fir terpenes, in some aspects preserve gene expression profile of older cells characteristics *in vitro* close to characteristics of younger cells. Abisil also induced expression of a range of anti- and pro-tumorigenic genes [16]. Other *in vitro* and *in vivo* studies have demonstrated anti-tumorigenic and anti-angiogenic effect of Abisil [17]. In the present study, we investigated the effect of Abisil on cell survival and metabolic rate, the processes of auto- and mitophagy, as well as genome-wide gene expression.

## MATERIALS AND METHODS

### Reagents and cell treatment

Abisil in the form of resin was kindly provided by Initium-Pharm LLC (Moscow, Russia; https://initium-pharm.com/). Abisil was dissolved in DMSO to obtain a stock for treatment of cell cultures.

All cells were maintained in Dulbecco's modified Eagle's medium (DMEM)(PanEco, Russia) supplemented with 10% fetal bovine serum (FBS, Biosera) and 50 U/ml penicillin and 50 μg/ml streptomycin (PanEco) in a humidified atmosphere containing 5% CO_2_. Immediately prior to treatment, Abisil stock was diluted in serum-free DMEM; treatment was performed in DMEM containing 5% FBS when cells were 50-70% confluent. Negative controls were treated with an equal amount of DMSO diluted in DMEM supplemented with 5% FBS.

### Oxidative stress assays

MRC5-SV40 cells were seeded at a density of 4×10^4^ cells/well in 12-well plates. Next day, Abisil treatment was performed in a wide range of concentrations (6-200 μg/ml). After 16 hours, cell oxidative stress was induced by treatment with 1 μM of rotenone (Sigma-Aldrich, USA) for 1.5 hours. After that, the cells were stained with 10 μM DHE (dihydroethidium, Sigma-Aldrich) for 35 min at 37° C. The cells were trypsinized and resuspended in PBS (PanEco) supplemented with 2% FBS. Before measurement cells were stained with 1 μg/ml of DAPI (Sigma-Aldrich) as a viability assay. Fluorescence was detected using a BD LSR Fortessa cytofluorimeter (Beckman Dickinson). The analysis was performed using Flowing Software 2.0 (Perttu Terho, Turku Center for Biotechnology). The results were based on three independent experiments, each with three biological replicates.

### Mitochondrial potential assessment

MRC5-SV40 and LECH-4 cells were seeded at a density of 4×10^4^ cells/well in 12-well plates. Next day, Abisil treatment was performed with a wide range of concentrations (10-80 μg/ml). After 16 hours, the cells were stained with 100 nM and 200 nM of TMRM (Thermo Fisher Scientific, USA), respectively, for 30 min at 37° C. 10 μM of CCCP (Sigma-Aldrich) was used as positive control. Before measurement cells were stained for viability with 1 μg/ml of DAPI (Sigma-Aldrich). Fluorescence was detected using a BD LSR Fortessa cytofluorimeter (Beckman Dickinson). The analysis was performed using Flowing Software 2.0 (Perttu Terho, Turku Center for Biotechnology). The results were based on three independent experiments with three biological replicates.

### Autophagy and mitophagy assays

Autophagy was assessed on a panel of cell lines stably expressing mCherry-GFP-LC3 reporter frame. Treatment with Abisil was carried out in concentration 50 μg/ml for 16 hours. Rapamycin (R0395, Sigma-Aldrich) was used as a positive control.

To detect mitophagy in MRC5-SV40 and LECH-4 cells, concentrations of 50 μg/ml of Abisil were used for treatment for 24 hours. After incubation, the cells were stained with 200 nM of MitoTracker Red CMXRos (Thermo Fisher Scientific) and 100 nM of LysoTracker Green (Thermo Fisher Scientific) for 20 minutes at 37° C.

Confocal microscopy was performed on the Nikon A1+MP confocal imaging system using an Apo TIRF 60x/1.49 oil DIC objective (numerical aperture=1.49; Nikon Japan), Apo LWD 40x/1.15 S water immersion objective (numerical aperture=1.15; Nikon Japan). Images were scanned sequentially using 488- and 561-nm diode lasers in combination with a DM405/488/561/633-nm dichroic beam splitter. Differential Interference Contrast Imaging (DIC) microscopy was used to visualize cell contours. The images were analyzed with NIS-elements AR (Nikon Japan).

### Quantitative real-time PCR

To analyze the level of mitochondrial DNA, total cellular DNA was extracted from the cells using GeneJET Genomic DNA Purification Kit (Thermo Fisher Scientific) and subsequently quantified by qPCR. qPCRmix-HS SYBR kit (Evrogen) was used according to the manufacturer’s instructions, and the real-time PCR was performed on the Rotor-Gene Q Real-Time PCR system (Quigen). The real-time qPCR results were analyzed with 2^−ΔΔCt^ method traditionally used for gene expression studies. We used genomic sequence of B2M gene as an endogenous control. All primer pairs used for qPCR are presented in the Table 1.

**Table 1:** Primers used for quantitative real-time PCR to evaluate mitochondrial genome cope number change after Abisil treatment.

	**Gene name**	**Primer name**	**Primer sequence (5’-3’)**
1	*B2M*	B2M-for	TGCTGTCTCCATGTTTGATGTATC T
2		B2M-rev	TCTCTGCTCCCCACCTCTAAGT
3	mitochondrial DNA, intergenic spacer	Mito-G-for	CGCCTCACACTCATTCTCAA
4		Mito-G-rev	CAAGGAAGGGGTAGGCTATG
5	mitochondrial DNA, *MT-ND1* gene	ND1-for	CCCTACTTCTAACCTCCCTGTTCTTAT
6		ND1-rev	CATAGGAGGTGTATGAGTTGGTCGTA

### Measurement of mitochondrial function using the Seahorse XF-24 extracellular flux analyzer

MRC5-SV40 cells were cultured in DMEM medium (Gibco, Thermo Fisher Scientific) containing 4.5 g/L glucose, supplemented with 10% bovine serum (BioSera) and 2 mM glutamine (PanEco). 40 hours before the experiment, the cells were seeded on 24-well Seahorse XFe24 Cell Culture Microplate (Agilent Technologies,) at a density of 12 000 cells per well in 500 μl of growth medium without affecting comparison wells A1, B3, C4 and D6 and incubated at +37° C in a CO_2_ incubator with a relative humidity of 95% and a CO_2_ content of 5%. When the cells reached 45% of the monolayer (24 hours after seeding) they were treated with Abisil at a concentration of 50 μg/ml and incubated for 16 hours at +37° C in a CO_2_ incubator with a relative humidity of 95% and a CO_2_ content of 5%. On the day of the MitoStress Test experiment, the growth medium was removed, the cells were washed with warm DMEM medium without sodium bicarbonate and supplemented with 25 mM glucose, 2 mM sodium pyruvate (PanEko, Moscow, Russia) and 2 mM glutamine (PanEko, Moscow, Russia) (2 times 300 μl), after which the experiment medium (described above, 500 μl per well) was added and incubated for 45 minutes at +37° C in a non-CO_2_ incubator. The experiment medium was also added to the comparison wells. The following preparations were added to the ports of the Agilent Seahorse XF24 Sensor Cartridge prepared in accordance with the manufacturer's recommendations: Port A - 11 μM oligomycin (1 μM final concentration in well), Port B - 24 μM FCCP (2 μM final concentration in well) and Port C - 13 μM Rot/AA (1 μM final concentration in well). Then, the Oxygen Consumption Rates (OCR) were measured (3 measurements per injection) at +37° C on the Seahorse Bioanalyzer instrument. After the measurement, the experiment medium was removed, 25 μl of RIPA buffer was added and the protein concentration was measured using a Bradford Reagent (Sigma B6916). OCR measurements were normalized to the total protein level.

On the day of the GlycoTest experiment, the growth medium was removed, the cells were washed with warm DMEM w/o sodium bicarbonate and supplemented with 2 mM sodium pyruvate (PanEco) and 2 mM glutamine (PanEco; 2 times 300 μL), added experiment medium (500 μl per well) and incubated for 30 minutes at +37° C in a non-CO_2_ incubator. The experiment medium was also added to the comparison wells. The following preparations were added to the ports of the Agilent Seahorse XF24 Sensor Cartridge prepared in accordance with the manufacturer's recommendations: Port A - 275 mM Glucose (25 mM final concentration in well), Port B - 12 μM oligomycin (1 μM final concentration in well) and Port C - 650 mM 2-deoxy-D-glucose (50 mM final concentration). Then, the Extracellular Acidification Rate (ECAR) was measured (3 measurements per injection) at +37° C on the Seahorse Bioanalyzer instrument. After the measurement, the experiment medium was removed, 25 μl of RIPA buffer was added, and the protein concentration was measured with a Bradford Reagent (Sigma B6916). ECAR measurements were also normalized to the total protein level.

### Evaluating cytotoxic effect of Abisil with MTS assay

MRC5-SV40 cells were seeded at a density of 5×10^3^ cells/well in a 96-well plate and cultured in 100 μl of DMEM supplemented with 5% FBS at 37° C. 24 hours after seeding cells were treated with Abisil (0; 9; 11; 15; 25; 50; 93; 125, 147 and 186 μg/ml) within 24 hours at 37° C. The level of cell metabolic activity was evaluated using the MTS assay (CellTiter 96 AQueous Non-Radioactive Cell Proliferation Assay (MTS), Promega) in accordance with the manufacturer's protocol. The optical density of the stained substance at 590 nm, proportional to the metabolic rate of viable cells, was measured using a plate reader (Tecan). Then, the IC50 values were calculated by the regression analysis, as well as the concentrations at which the most intense cellular metabolism was observed.

### Western blotting

MRC5-SV40 cells were cultured on 35 mm Petri dishes with confluency of 50%, treatment with Abisil was carried out in 5% DMEM supplemented with 5% FBS at 37° C overnight. After incubation the cells were washed with cold PBS and lysed in 200 μl of Reporter Lysis Buffer (Promega) containing a Roche protease inhibitor cocktail (# P8349, Sigma-Aldrich), according to the manufacturer's instructions. Lysates were centrifuged for 15 minutes at 12,000 g; total protein content in the protein supernatant was measured by the Bradford assay. Proteins were separated by Laemmli's electrophoresis in a 12% SDS-polyacrylamide gel, electrotransfer to a PVDF membrane (Amersham Hybond P 0.45 μm, Amersham Biosciences, GE Healthcare) was performed in Towbin transfer buffer (25 mM Tris, 192 mM glycine, 20% (v/v) ethanol, pH 8.3). Membranes were blocked in a 4% solution of skim milk in PBST (PBS with 0.05% Tween 20) for 1 hour at room temperature. Incubation with primary and secondary (goat anti-rabbit IgG-HRP, sc-2004, Santa Cruz Biotechnology) antibodies was performed overnight at 4° C and for 1 hour at room temperature, respectively. Immobilon Western Chemiluminescent HRP Substrate (Merck-Millipore, Sigma-Aldrich) was used for detection; the chemiluminescence signal was determined using the Bio-Rad ChemiDoc MP Imaging System (Bio-Rad). Quantification of protein bands was performed using ImageJ software (NIH, Bethesda, MD, USA). The following primary antibodies were used: β-actin (D6A8, #8457, Cell Signaling Technology), LC3 (D11, #3868, Cell Signaling Technology).

### Transcriptomic analysis

### Sample preparation

MRC5-SV40 cells were plated on 60 mm Petri dishes (3×10^5^ cells per a dish) and cultured overnight at 37° C in DMEM supplemented with 10% FBS. Next day, treatment with Abisil was carried out with concentrations of 5 μg/ml and 50 μg/ml in DMEM with 5% FBS. Control samples were incubated in DMEM with 5% FBS and 0.025% DMSO. After 16 hours, the medium was removed and the cells were washed with PBS, followed by lysis using 500 μl of lysis buffer (MagNA Pure Compact RNA Isolation Kit, Roche). All treatment experiments were performed in triplicate.

### RNA isolation, preparation and sequencing of RNA-Seq libraries

Total RNA was extracted using the RNeasy Mini kit (Qiagen, Germany) according to the manufacturer's protocol. The quality and quantity of RNA was determined using an Agilent 2100 bioanalyzer (Agilent Technologies, USA). RNA samples with RINs above 9.0 were used to prepare libraries. Total RNA (2 μg) from each sample was used to prepare the mRNA library using the TruSeq RNA Sample Preparation Kit v2 Low Sample (LS) protocol (Illumina, USA) in accordance with the manufacturer's protocol. The cDNA libraries were sequenced (single end reads, 50 bp) using Illumina NextSeq 500 system in the "Genome" Center (EIMB RAS, Russia, http://www.eimb.ru/rus/ckp/ccu_genome_c.php).

### RNA-Seq data processing and differential expression analysis

Raw reads were checked for quality and trimmed using the FastQC and Trimmomatic [18] tools. The trimmed reads were mapped to the human reference genome (GRCh38.80, Ensembl) with splice-aware STAR mapper [19]. Next, 5’-to-3’ transcript read coverage distribution was evaluated using geneBody_coverage.py script from RSeQC package [20] in order to ensure the absence of 3’-tail bias. Read counting per gene was done using featureCounts tool from the Subread package [21]. The further analysis was performed in R environment using edgeR (identification of differentially expressed genes), biomaRt (annotation), topGO and clusterProfiler (GO, KEGG, Reactome enrichment analyses), pathview (visualization) and other packages.

### Proteomic analysis

### Sample preparation

The MRC5-SV40 line was also used for panoramic proteomic analysis. 3×10^5^ cells were plated on 60 mm Petri dishes. After a night of incubation in the complete medium 16-hour treatment with Abisil at concentration of 50 μg/ml in DMEM medium with 5% FBS was performed. Control samples were treated with DMEM with 5% FBS and 0.025% DMSO. After 16 hours the medium was removed and the cells were washed twice with PBS, then the cells were removed from the dishes in 800 μl PBS using a scraper.

The sample preparation for proteomic analysis was performed using Pierce Mass Spec Sample Prep Kit (Thermo Fisher Scientific, MA, USA) [22]. In addition, Thermo Scientific Pierce Digestion Indicator from the same kit, was used to assess the digestion reproducibility for multiple samples. For sample preparation 1×10^6^ cells (100 μg of protein) were lysed using modified radioimmunoprecipitation assay buffer (RIPA), which was followed by reduction step using 2M dithiothreitol for 45 min at 50° C. For alkylation step iodoacetamide was added to the sample (final volume 50 mM) and incubated at room temperature for 20 min. Immediately after incubation 4 volumes (460 μl) of pre-chilled (-20° C) 100% acetone was added to samples. Two-step enzymatic protein digestion was performed using Lys-C (enzyme-to-substrate ratio = 1:100, 37° C for 2 hours) and trypsin (enzyme-to-substrate ratio = 1:50, 37° C for 17 hours). To remove the digestion buffer samples were dried at the SpeedVac and resuspended in 0.1% TFA buffer. Protein concentration was measured using bicinchoninic acid assay (BCA assay) [23].

### LC-MS/MS

LC-MS/MS was performed using an TIMS TOF Pro mass spectrometer (Bruker Daltonics, USA) combined with an UltiMate 3000 nano LC system (Thermo Fisher Scientific, MA, USA). The amount of loaded sample was 100 ng per injection. The tryptic peptide fraction (injection volume 1 μL) was analyzed in triplicate on a Dionex UltiMate 3000 Nano HPLC System (Thermo Fisher Scientific, MA, USA) coupled to a TiMS TOF mass spectrometer (Bruker Daltonics, Bremen, Germany) using a captive spray ion source (positive ion mode, 1600 V) (Bruker Daltonics, Bremen, Germany). HPLC separation was performed on a C18 capillary column (25cm x 75μm 1.6μm) (Ion Optics, Parkville, Australia) by gradient elution. The mobile phase A was 0.1% formic acid diluted in water and mobile phase B was 0.1% formic acid diluted in acetonitrile. The separation was carried out by a 120-min gradient from 4% to 90% of phase B at a flow rate of 0.4 μL/min. Mass-spectrometric measurements were carried out using Parallel Accumulation Serial Fragmentation (PASEF) acquisition method. The measurements were carried out in m/z range from 100 to 1700. The range of ion mobility include a value from 0.60 – 1.60 Vs/cm2 (1/k0). The number of precursor ions per one PASEF cycle (1.2 sec) was 10. Low abundant precursor ions can be analyzed multiple times during PASEF cycle.

### Protein identification and quantification

For label-free analysis the obtained MS spectra were processed with MaxQuant software against the SwissProt 2018 database [24]. Oxidation of methionine and acetylation of N-terminal were selected as variable modification. Minimal length for peptide identification was seven amino acids and at least one peptide should be unique for a protein. False discovery rate (FDR) threshold for proteins and peptides was set 0.01. Additionally, match between the runs option were used.

### Identification of altered proteins

Protein quantification was carried out using label free quantitation intensities (LFQ) based on relative protein quantification across all samples and represented by a normalized intensity profile that is generated according to the algorithms described by Cox, J. et al. [25]. Statistical processing of proteomic data was performed in R environment. Two samples Welch's t-test was applied to identify the significantly altered proteins between treated and control groups. Correlation between proteins composition in technical runs was estimated using Pearson’s correlation analysis for log-transformed abundance values (the values of correlation coefficient were about 0.9).

### Pathway enrichment analysis

Differentially regulated proteins were divided into several groups for the pathway enrichment analysis: (1) upregulated: FDR < 0.05 (Welch's t-test), two-fold ore greater fold change; (2) downregulated: FDR < 0.05, two-fold ore greater fold change; (3) a group of marginally altered proteins with FDR < 0.05 and fold change less than two-fold. Each protein group was analyzed separately for GO enrichment. Protein interaction network analysis was done using STRINGdb [26]. The results of GO enrichment analysis was grouped by semantic similarity using the REVIGO tool [27].

### Data availability

Transcriptome sequencing data are available at NCBI SRA portal (BioProject identifier PRJNA642179).

## RESULTS

### Cytotoxicity of different concentrations of Abisil

**Figure 1:**
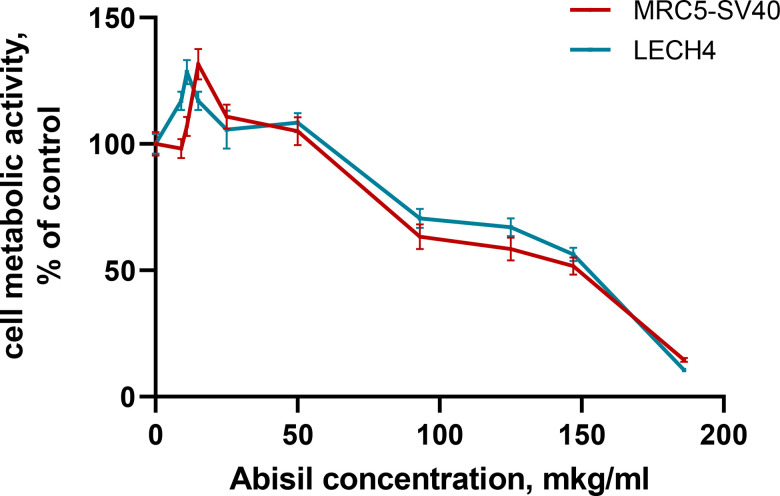
Assessment of the metabolic activity of cells treated with various concentrations of Abisil (MTS assay).

The effect of 48-hour treatment with Abisil on the metabolic activity of cells was studied using the MTS assay on human embryonic lung fibroblast lines (MRC5-SV40 and LECH-4). Abisil demonstrated significant dose dependence (Figure 1). The calculated IC50 values were 149.3 ± 0.67 for MRC5-SV40 and 152.6 ± 0.52 μg/ml for LECH-4, respectively (Table 2). In addition, both cell lines showed a significant increase in metabolic activity (p-value <0.001) when treated with the low drug concentrations (9—15 μg/ml).

**Table 2:** Cytotoxic effect of Abisil.

**Cell line**	**IC50, μg/ml**	**Metabolic activity normalized to control, %**	**Concentration of the drug, causing the maximum metabolic rate, μg/ml**	**Metabolic activity normalized to control, %**
Embryonic lung fibroblasts immortalized with SV40 (MRC5-SV40) virus	149.3 ± 0.7	50	15	131.5
Primary embryonic pulmonary fibroblasts (LECH-4)	152.6 ± 0.5	50	11	128.2

Evaluating cell metabolic activity with MTS assay.

### Antioxidant activity of Abisil

After a series of experiments, DHE staining method (described in the materials and methods) was chosen as optimal approach to evaluate the levels of ROS after treatment with Abisil in a wide concentration range. This method demonstrated good reproducibility (range of variation 10-25%).

**Figure 2:**
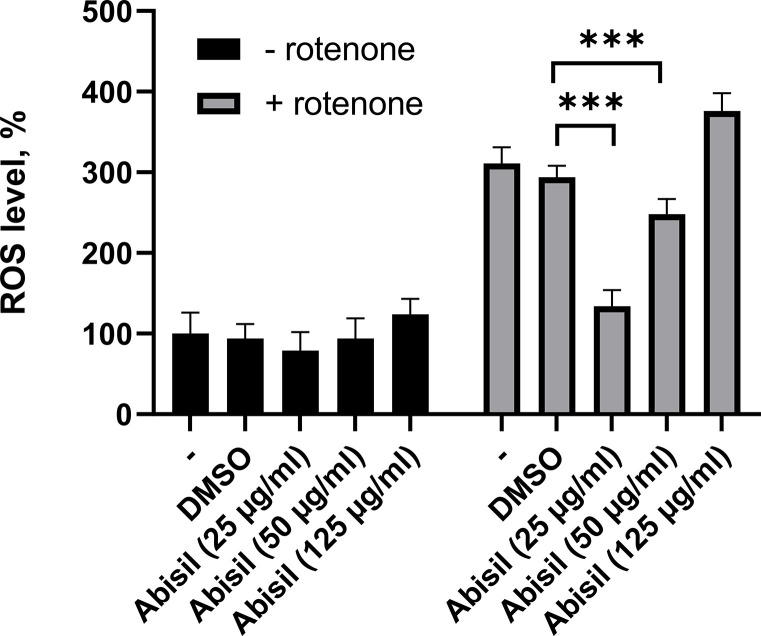
**ROS levels assessment in MRC5-SV40 cells treated with various concentrations of Abisil.** ***p < 0.001 (*t*-test).

The diagram on the Figure 2 shows the ROS levels assessment results after a 16-hour treatment with Abisil in the range of concentrations 25-125 μg/ml.

The results demonstrate that the most intense decrease in ROS after induction by rotenone is observed for concentrations of 25 and 50 μg/ml (Figure 2). It is worth noting that there is a significant prooxidant effect at higher concentrations of the drug (125 μg/ml). There is also a tendency towards a decrease in ROS in the absence of inductor.

### Abisil causes decrease in mitochondrial potential

**Figure 3:**
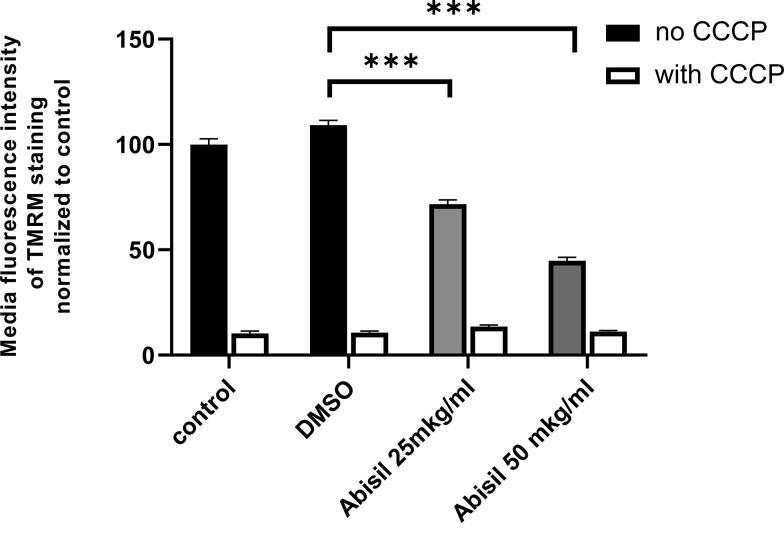
**Abisil causes decrease in mitochondrial potential in immortalized MRC5 cells.** MRC5-SV40 cells were incubated with Abisil (25 mkg/ml and 50 mkg/ml) overnight and stained with TMRM, and fluorescence of the stain was measured in PE channel. The black bars represent Abisil-treated cells; the grey bars represent control cells. ***p < 0.001 (*t*-test).

As Abisil downregulates genes encoding for subunits of the respiratory complex I (according to RNA-Seq data; described below; Supplementary Figure 2), it was tested if Abisil might cause the decrease of mitochondrial potential. Indeed, Abisil treatment induced more than 2 times-fold drop of mitochondrial potential (p-value < 0.01, Mann-Whitney U-test; Figure 3). As a positive control, we used carbonyl cyanide chlorophenylhydrazone (CCCP). This is a lipid-soluble weak acid, mitochondrial toxin and a potent mitochondrial uncoupling agent. It increases the proton permeability across the mitochondrial inner membrane, thus dissipating the transmembrane potential and depolarizing the mitochondria.

### Abisil treatment results in intense autophagy after 24 hours

**Figure 4:**
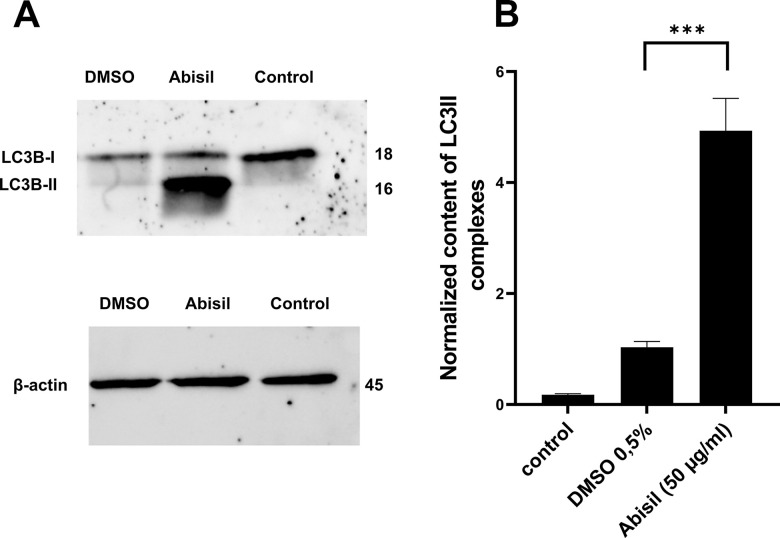
**Effect of Abisil pretreatment on LC3II accumulation in MRC5-SV40.** (**A**) Western blot analysis of LC3 protein. (**B**) densitometric analysis results of LC3II normalized to endogenous control (beta-actin). ***p < 0.001 (*t*-test).

Autophagy was assessed by the amount of LC3II in MRC5-SV40 cells by western blotting (Figure 4A). Figure 4B shows the results of LC3II densitometric analysis with respect to internal control (beta-actin) which are presented as mean ± standard deviation calculated from three independent experiments. There is a significant increase in the content of LC3II complexes (more than 10-fold) when treated with Abisil at concentration of 50 μg/ml, which shows effective stimulation of autophagy.

**Figure 5:**
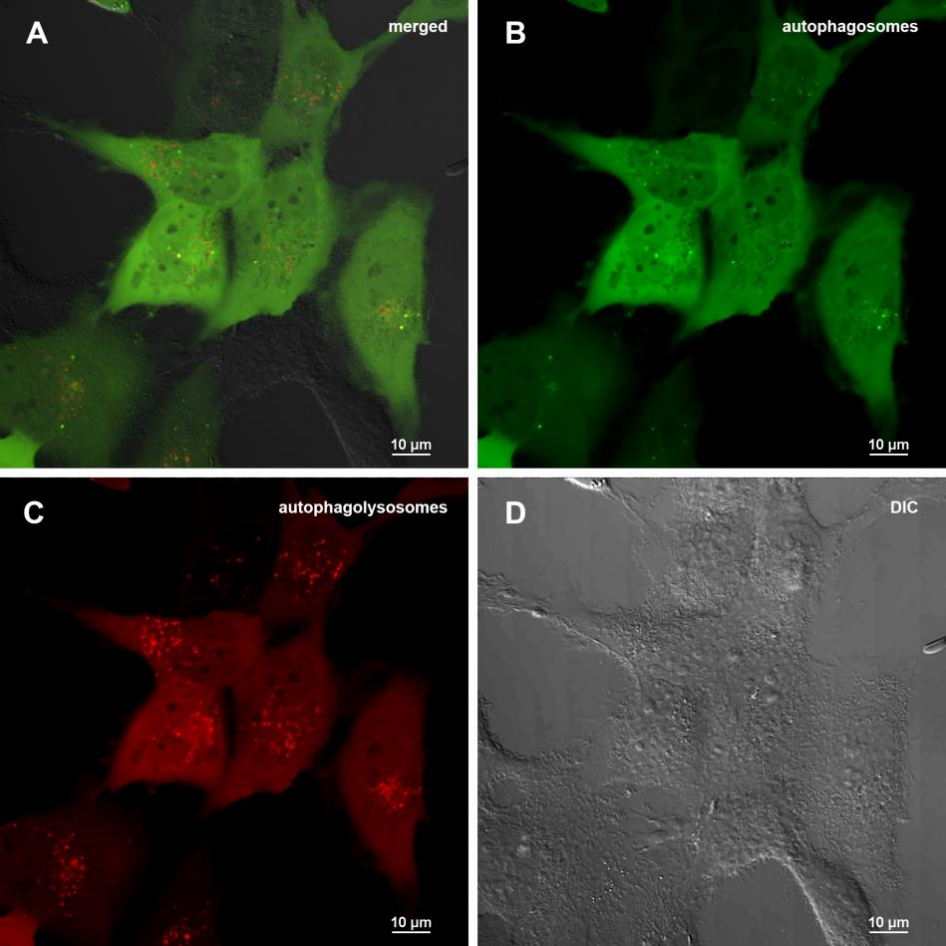
**Assessment of autophagy in MRC5-SV40 cells carrying GFP-LC3-mCherry reporter (control sample) when treated with DMSO 0.5% after 24 hours.** (**A**) merge of red, green fluorescence channels and bright field, (**B**) green fluorescence channel, (**C**) red fluorescence channel, (**D**) bright field.

**Figure 6:**
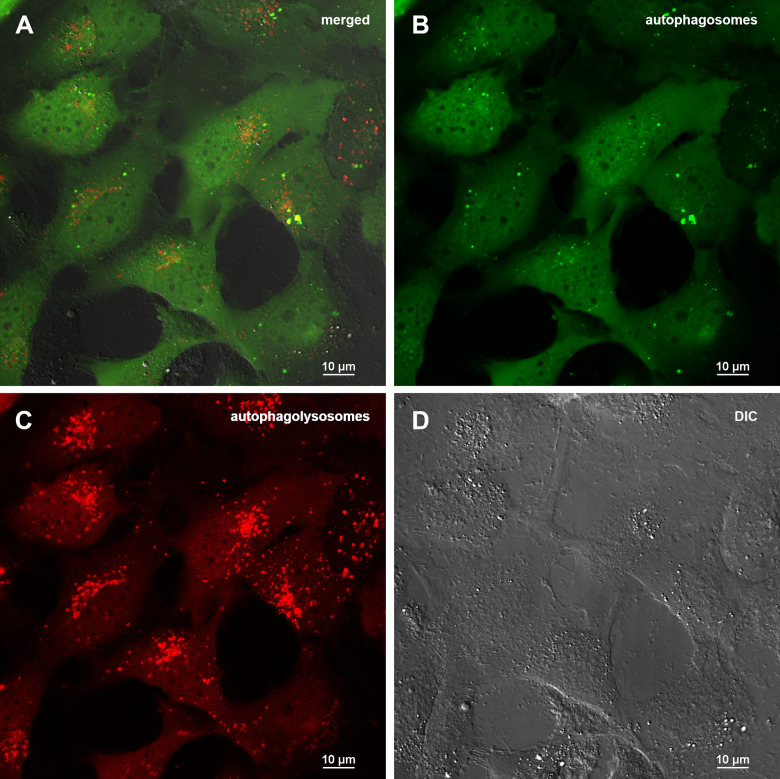
**Assessment of autophagy in MRC5-SV40 carrying GFP-LC3-mCherry reporter when treated with 50 μg/ml Abisil after 24 hours.** (**A**) merge of red, green fluorescence channels and bright field, (**B**) green fluorescence channel, (**C**) red fluorescence channel, (**D**) bright field.

**Figure 7:**
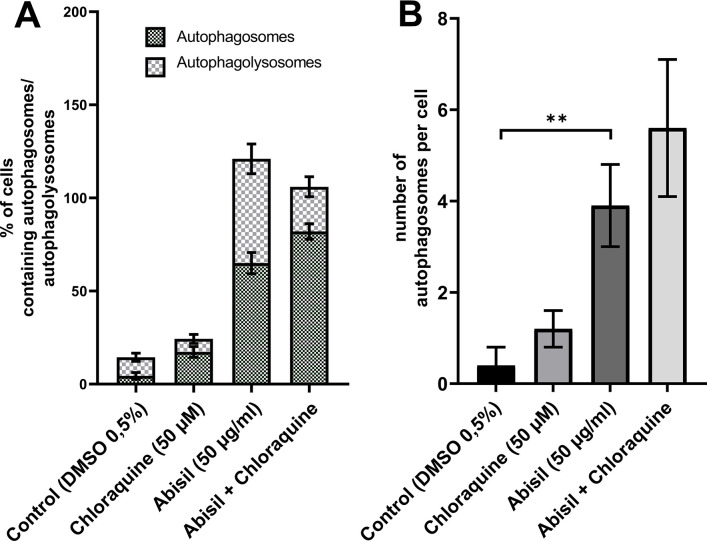
**Assessment of autophagy in MRC5-SV40 carrying GFP-LC3-mCherry reporter when treated with Abisil (50 μg/ml) in presence or absence of chloroquine (50 μM) after 24 hours.** (**A**) percentage of cells containing autophagosomes, (**B**) number of autophagosomes per cell. **p < 0.01 (*t*-test).

To carry out an accurate quantitative assessment of the effectiveness of autophagy, a 3D reconstruction of a set of fields was carried out using a z-stack approach on a confocal microscope. 20 fields were used for each of sample type: control (0.5% DMSO), treated with Abisil 50 μg/ml, treated with chloroquine 50 μM and treated with both Abisil and chloroquine). An example of the images obtained is shown below (Figures 5, 6). Data on the number of large autophagosomes are shown in the diagram (Figure 7). As you can see below, autophagy is induced efficiently in 24 hours after treatment.

### Abisil does not increases the level of mitophagy and doesn’t affect the number of mtDNA copies

**Figure 8:**
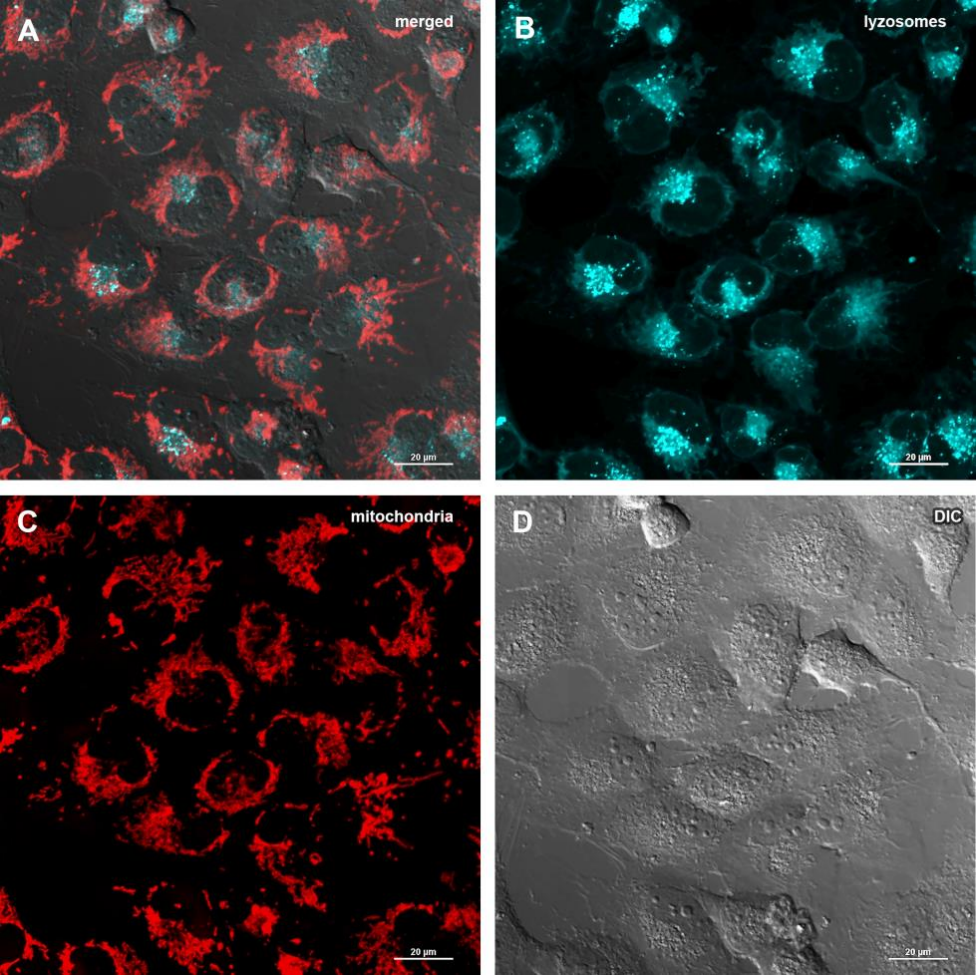
**Assessment of mitophagy in MRC5-SV40 cells when treated with DMSO 0.5% for 24 hours.** (**A**) merge of red, green fluorescence channels and bright field, (**B**) green fluorescence channel, (**C**) red fluorescence channel, (**D**) bright field.

**Figure 9:**
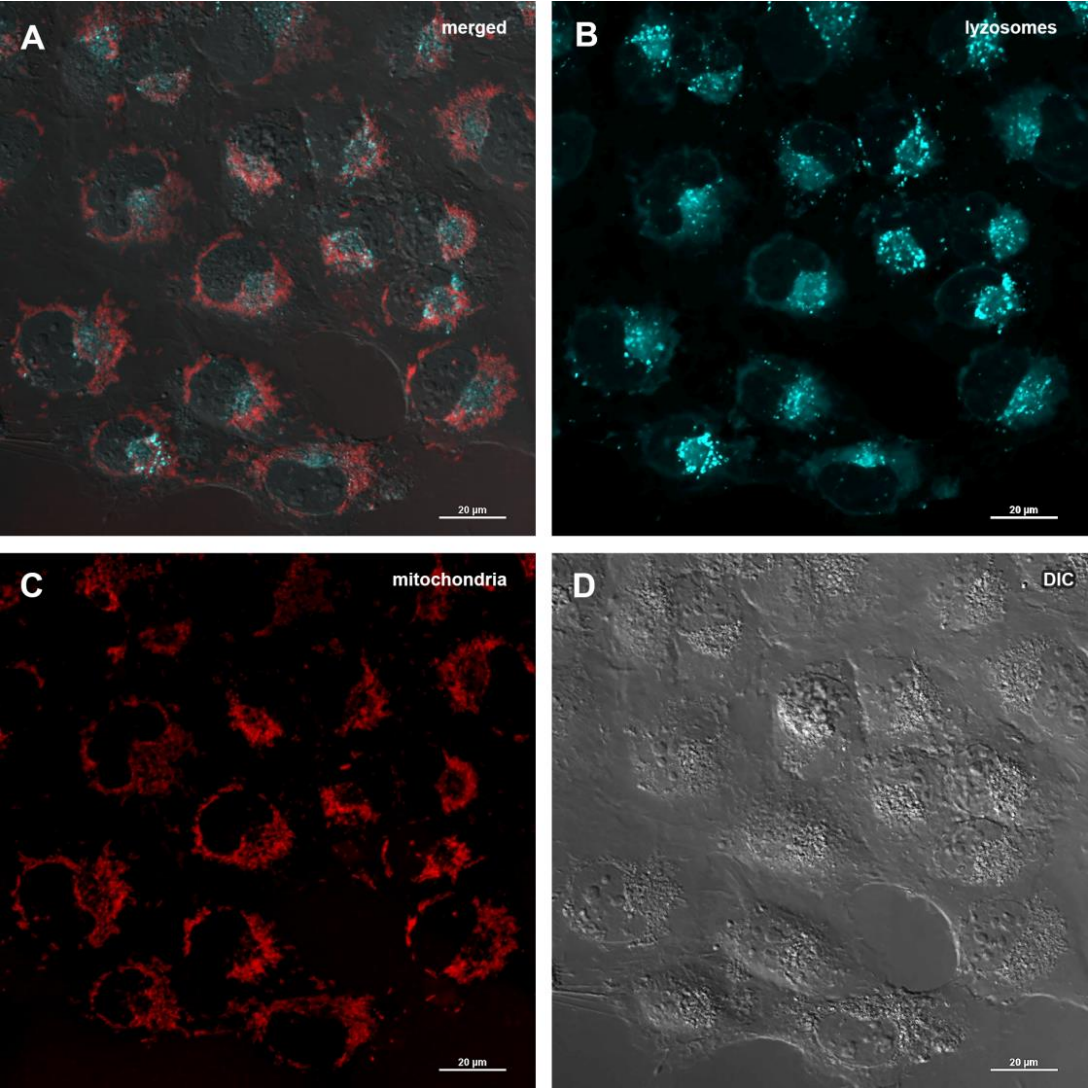
**Assessment of mitophagy in MRC5-SV40 cells when treated with Abisil at concentration of 50 μg/ml for 24 hours.** (**A**) merge of red, green fluorescence channels and bright field, (**B**) green fluorescence channel, (**C**) red fluorescence channel, (**D**) bright field.

The decrease of mitochondrial potential caused by Abisil treatment might imply that Abisil induces mitophagy [28–30]. In order to verify this hypothesis, MRC5-SV40 cells were treated with Abisil and tested whether Mitotracker Red colocalized with Lysotracker Green (Figures 8, 9). There was an increase of the number of phagolysosomes, but they did not colocalize with Mitotracker Red indicating that Abisil promotes autophagy but does not specifically induce mitophagy in SV40 transformed MRC5. The same treatment was conducted on LECH-4 cells with the same result (data not shown).

**Figure 10:**
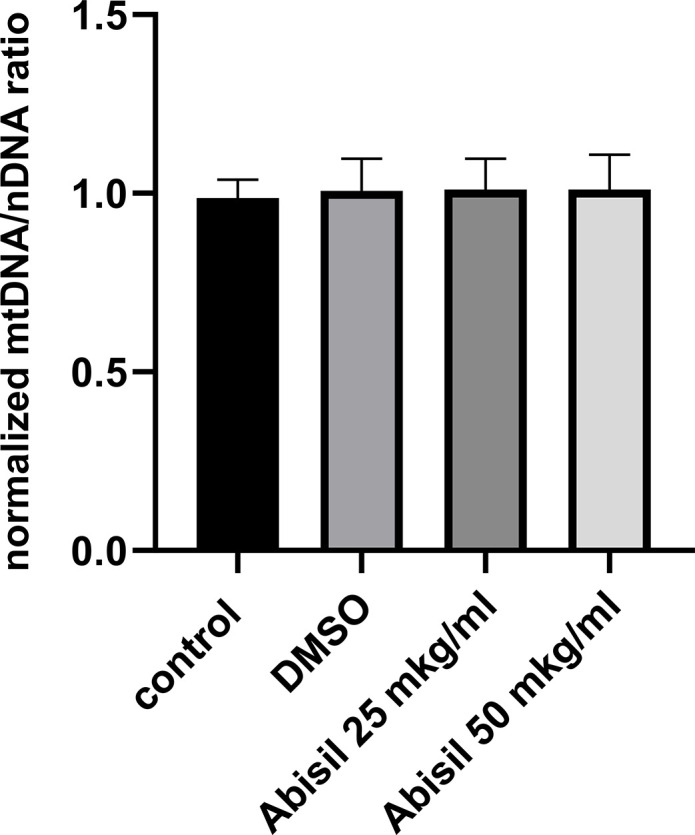
**Relative mitochondrial copy number determined by qPCR in MRC5-SV40 cells, untreated (control), treated with DMSO or Abisil (16 hours after treatment).** The mtDNA/nDNA copy number ratio was normalized to DMSO treated samples.

Changes in mitochondrial potential detected in previous experiments suggested to test also the copy number of mitochondria DNA. As can be seen from the diagram in Figure 10, the amount of mitochondrial DNA did not change at all.

### Abisil induce changes in cell energy metabolism

**Figure 11:**
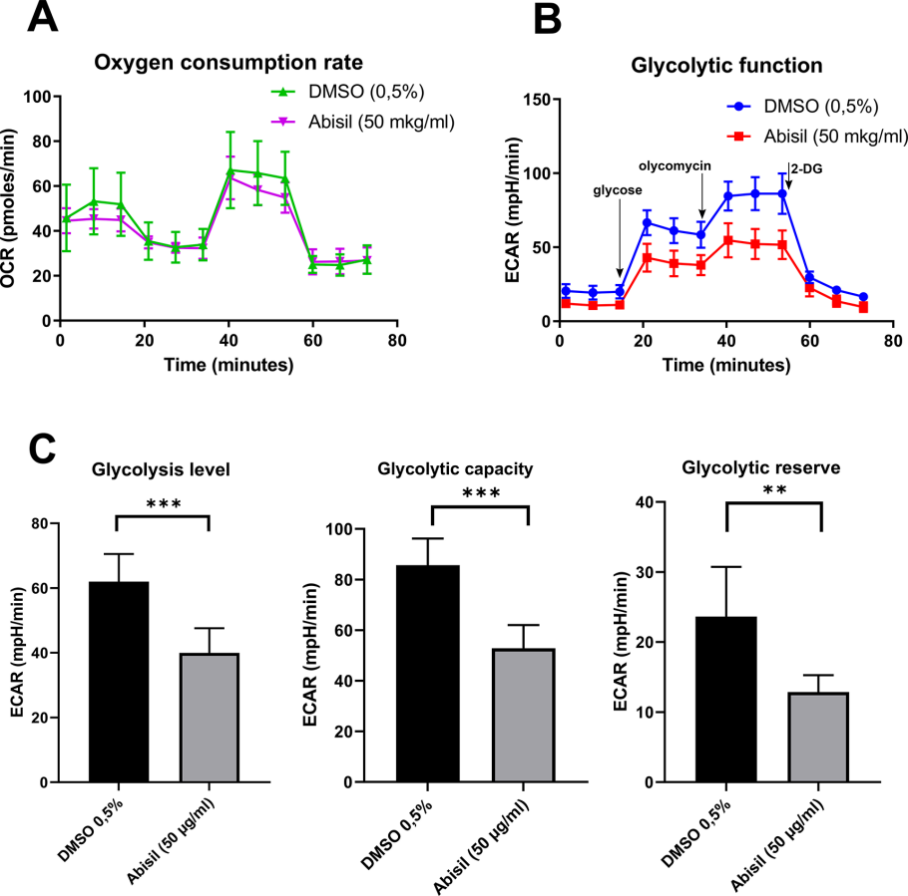
**Changes in mitochondrial respiration and glycolysis level of MRC5-SV40 cells after Abisil treatment.** (**A**) After 16 hours of Abisil treatment, mitochondrial respiration reflected by the level of oxygen consumption rate (OCR) in control (DMSO 0.5%) or Abisil (50 μg/ml) groups (n = 4 per group), following the injection of oligomycin, FCCP and antimycin A/rotenone. (**B**) the extracellular acidification rate (ECAR) of the medium following injection of glycose, oligomycin and 2 desoxyglucose. (**C**) The rates of glycolysis level, glycolytic capacity and glycolytic reserve (n = 4 per group). Bars represent mean values with SD, **p < 0.01, ***p < 0.001 (*t*-test). Glycolytic level – the basal rate of conversion of glucose to pyruvate/lactate; glycolytic capacity – the maximal rate of glycolysis that can be rapidly achieved by a cell; glycolytic reserve – the difference between the glycolytic capacity and the basal glycolytic rate.

Using the Seahorse XF-24 extracellular flux analyzer, we analyzed changes in mitochondrial respiration and glycolysis rates after between Abisil-treated and control cells. The results are presented at the Figure 11. While the rate of oxygen consumption drops slightly, the glycolytic activity is significantly reduced. Thus, we see that both the current activity of glycolysis and its maximum rate, possible for the cell (glycolytic capacity), decrease proportionally.

### Transcriptomic changes induced by Abisil treatment

We performed RNA-Seq analysis for MRC5-SV40 cells treated with Abisil at concentrations 5 and 50 μg/ml as well as control cells (Supplementary Tables 1–4). Taken at both concentrations, Abisil treatment induced vast transcriptomic shift spanning several thousand genes. The effect of treatment with both concentrations was generally similar (see the Supplementary Figure 3). Surprisingly, at a concentration of 5 μg/ml, the effect was even more pronounced. In this case, 2075 genes passed FDR < 0.05 threshold and had at least 1.5-fold expression level changes. We noted a predominant decrease in expression. So, among the top 200 differentially expressed genes (DEGs), 189 were downregulated. The lists of DEGs between 5 and 50 μg/ml significantly overlapped. The spectra of enriched Gene Ontology terms also overlapped. At both concentrations of Abisil, the lists of downregulated DEGs were strongly enriched with genes involved in cell cycle and related processes. In addition, we noted overrepresentation of genes involved in mRNA processing, protein folding, fatty acid elongation, NIK/NF-κβ and WNT signaling, antigen processing and presentation, cellular response to hypoxia.

Among biological processes, participants of which were overrepresented in upregulated genes, we noticed Notch signaling pathway, positive and negative regulation of apoptotic process, heterocycle catabolic process and other processes. We noticed bidirectional expressional changes of genes encoding for ribosomal proteins, but it is hard to say about the prevalence of any direction.

**Figure 12:**
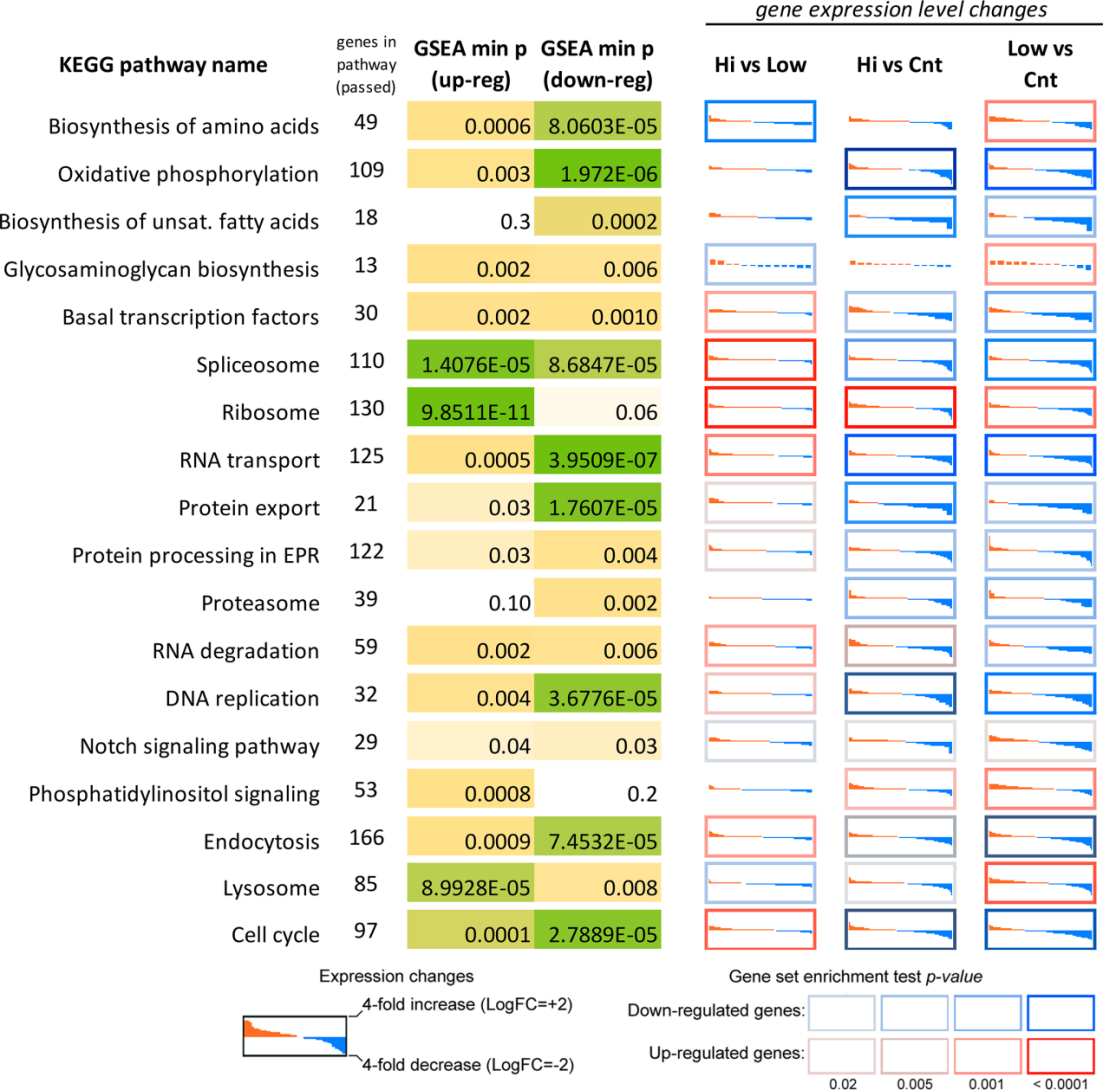
**Differential expression profiles of genes participating mostly affected KEGG pathways according to the RNA-Seq data for MRC5-SV40 cell line treated with Abisil.** Each cell demonstrates the sorted expression level log fold changes after Abisil treatment (red – upregulation, blue – downregulation) for genes participating a current KEGG pathway (vertical axis range is from 4-fold downregulation to 4-fold upregulation). Cell borders indicate whether a pathway is enriched with up- (red border) or downregulated (blue border) genes. *GSEA min p (up/down-reg)* – minimal p-value in a gene set enrichment analyses (GSEA; Fisher’s exact test) for the pathway. *Hi vs Low* – comparison of cells treated with 50 and 5 μg/ml; *Hi vs Cnt* – comparison of cells treated with Abisil 50 μg/ml and non-treated cells; *Low vs Cnt* – comparison of cells treated with Abisil 5 μg/ml and non-treated cells.

Interestingly, KEGG enrichment analysis revealed significant overrepresentation of downregulated DEGs among participants of oxidative phosphorylation, DNA replication process, basal transcription factors, spliceosome subunits, proteasome subunits (for both Abisil concentrations). Figure 12 shows the transcriptomic effect of Abisil treatment on genes involved in several mostly affected KEGG pathways. For a more detailed info, see the Supplementary Table 9.

The comparison of samples treated with different Abisil concentrations (5 and 50 μg/ml) revealed 1281 genes that passed FDR < 0.05 threshold, but only 305 of them had 1.5-fold or greater expression level changes. The lists of DEGs demonstrated enrichment in genes involved in the regulation cell proliferation and growth, cytoskeleton organization, RNA splicing, cell metabolism, including biosynthesis of amino acids, chemotaxis and cell adhesion, extracellular matrix organization, ion transport. The complete results of GO enrichment analyses are presented in the Supplementary Tables 5–8.

Mainly, the effect of Abisil on biological processes and cellular pathways can be described as: 1) suppressing genes participating in oxidative phosphorylation, DNA replication (especially at low Abisil concentrations), mRNA transcription and splicing, RNA transport, fatty acid biosynthesis, protein export, proteolysis; 2) activating phosphatidylinositol signaling, cytosolic DNA-sensing pathway, PPAR signaling pathway, various pathways involved in the immune response; 3) bidirectional impact on cell cycle (predominantly, downregulation) and endocytosis.

**Figure 13:**
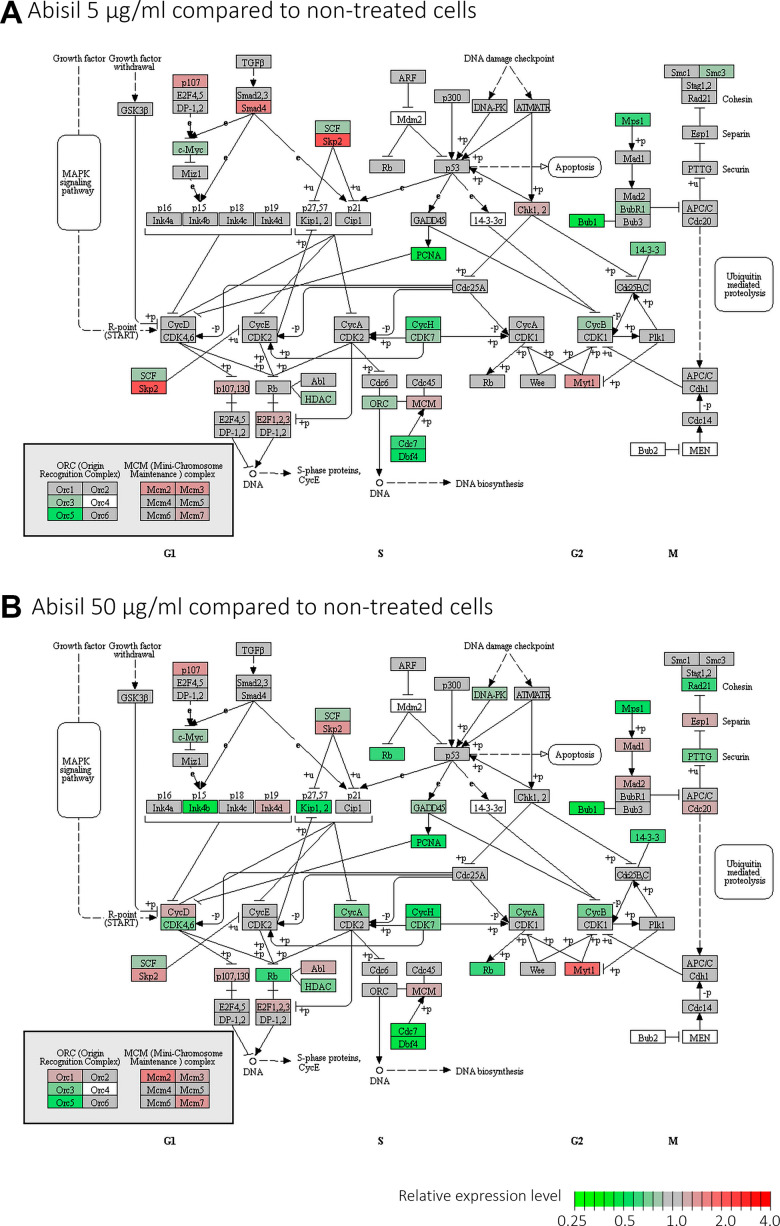
**Diagram illustrating expression changes of genes participating cell cycle signaling pathway (KEGG) after Abisil treatment (MRC5-SV40 cell line):** 5 μg/ml (**A**) and 50 μg/ml (**B**). Green – downregulation, red – upregulation.

Two pathways, cell cycle and oxidative phosphorylation, require special attention (Figure 13 and Supplementary Figure 2). The pattern of transcriptomic impact of Abisil on genes participating in cell cycle is ambiguous, although in general downregulation strongly prevails. This is especially evident when cells were treated with Abisil in low concentration (Figure 13). One can see a decrease in the expression levels of cyclins *CycA*, *CycB*, *CycH*, as well as cyclin-dependent kinases *CDK4*, *CDK6*, *CDK7* and other genes that contribute to cell cycle progression. However, against this background, a decrease in the expression levels of several genes involved in the negative regulation of the cell cycle (*Kip1*, *Kip2*, *PCNA*, *Rb*, etc.) is also noted.

Regarding the oxidative phosphorylation pathway, it is remarkably, that Abisil induces not only reducing mitochondrial potential itself (see the Figure 3), but also reduces the expression of genes participating oxidative phosphorylation. (Supplementary Figure 2) Taken at both concentrations, it causes a decrease in the expression mainly for genes of the mitochondrial genome: NADH dehydrogenase (ND1-6) and cytochrome C oxidase (*COX1-3*). Expression levels of genes from the cell nuclear genome, whose products are involved in oxidative phosphorylation (*NDUFA*, *NDUFB*, succinate dehydrogenase, ATPase), are altered to a much lesser extent. Both up- and downregulations are noted mainly for treatment with high concentration of Abisil (Supplementary Figure 2).

Worth noting, Abisil treatment results in predominant downregulation of many genes encoding for kinases and kinase-associated proteins (KKPs; containing “kinase” keyword in a gene name). Among top 50 differentially expressed KKPs after treatment with Abisil at 5 μg/ml, 35 are downregulated, and the average log2(fold change) for all these 50 genes is -0.45. The concentration 50 μg/ml gives less pronounced result.

Thus, such effect is more pronounced for low concentration of the drug (5 μg/ml). Moreover, we noticed that 27 of 30 (90%) KKPs downregulated after 50 μg/ml treatment (FDR < 0.05) lies within the list of 70 downregulated KKPs after 5 μg/ml treatment (FDR < 0.05). These 27 KKPs are: *BUB1, MAP2K4, MNAT1, LYN, AXL, JAK1, CSNK2A2, AK6, TAB2, SNRK, AK3, CLK1, DYRK1A, CDKN3, CDK6, FASTKD3, SH3KBP1, PRKACB, MOB1B, TTK, PXK, MAP3K20, PRKRA, RPS6KC1, PRKD3, NRK, PIK3R1*.

**Figure 14:**
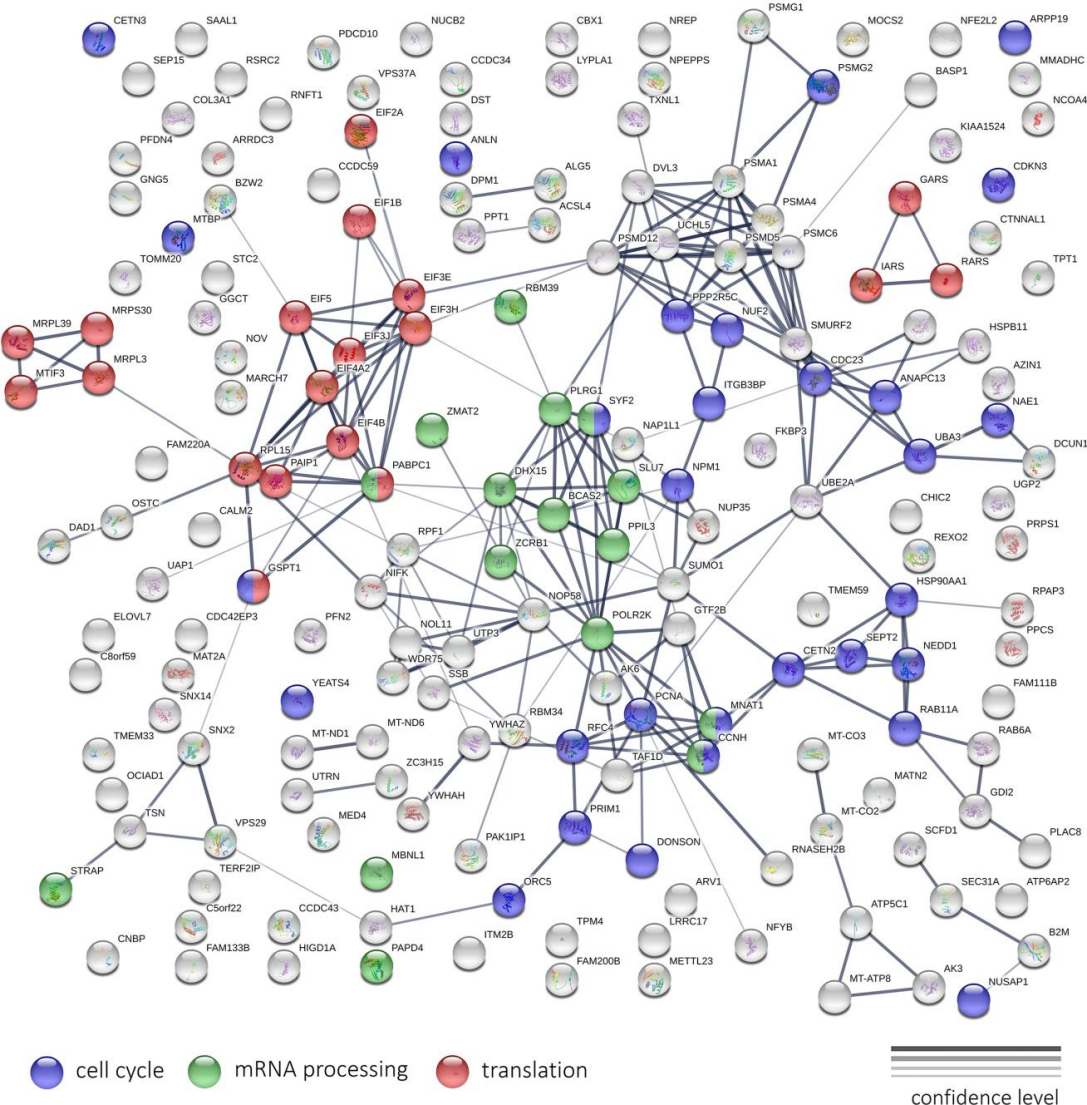
**The interaction network of protein products encoded by top 200 genes downregulated after Abisil treatment, according to RNA-Seq data (5 μg/ml; MRC5-SV40 cell line).** The width of the connecting lines indicates the confidence level of protein interaction (best – mentions in curated databases, experimental data for human; worst – found interacting putative homologs in other organisms).

Figure 14 illustrates the results of the protein-protein interaction network analysis for top 200 genes that are downregulated after Abisil treatment (5 μg/ml). We can see several net modules, that are responsible for translation, mRNA processing, regulation of cell cycle and other activities. Even though Abisil stimulates autophagy (as demonstrated above), however, at the transcriptome level, we do not see a consistent and unidirectional change in the expression of genes involved in autophagy, mTOR and AMPK signaling.

### Analysis of panoramic proteomic sequencing results

Large-scale quantitative proteome profiling was performed to reveal differentially expressed proteins in MRC5-SV40 cells treated with Abisil (50 μg/ml) compared to non-treated ones. Total 4906 proteins have been detected either for treated or non-treated cells. As it was noticed for transcriptome profiling, we observed that Abisil treatment mainly induces downregulation of expression. 4002 proteins were detected in non-treated MRC5-SV40 cells and only 3068 in Abisil-treated (2164 proteins were detected in both conditions). The detailed data on the proteome profiling is presented in the Supplementary Table 10.

Gene Ontology, KEGG and Reactome overrepresentation analysis revealed that the list of top 300 down-regulated proteins is enriched with participants of cell cycle regulation, exocytosis, secretion. Both lists, top 300 down- and top 300 up-regulated proteins were highly enriched with participants of RNA processing, translation, mitochondrial organization, oxidative phosphorylation, cell metabolism, transport, gene expression regulation, histone modification, membrane trafficking, ER to Golgi anterograde transport, and many other biological processes.

Protein-protein interaction (PPI) networks for down- and up-regulated proteins is presented in the Supplementary Figures 4, 5. Both networks are highly enriched with PPIs (p < 10^-16^) according to the STRING database analysis results. We can see that proteins, participating in translation, mitochondrial organization, RNA processing, form compact subnets (marked with colors), whereas proteins involved in cell metabolism and intracellular transport are spread over the whole network.

To sum up the analysis, it is possible to conclude that the content of many proteins in the cell decreases (possibly, because of autophagy) after Abisil treatment. The expression of genes/proteins, involved in translation, mitochondrial organization, gene expression regulation, cell metabolism and transport, is significantly altered (either down- or upregulated).

It is remarkably that the content of such proteins as mTOR, RICTOR (Rapamycin-insensitive companion of mTOR), Cytochrome c, RBL1 (Retinoblastoma-like protein 1), Ras-related protein Rab-13, TGF-beta receptor type-1 (TGFBR1), Tumor necrosis factor receptor superfamily member 12A (TNFRSF12A), Cyclin-dependent kinase 9 (CDK9), Mitogen-activated protein kinases (MAPK14, MAP3K6, MAP3K2, MAPK8, MAPK3, MAP4K5, MAPK14) and Renin receptor (ATP6AP2) was significantly reduced (or even not detected by MS) after treatment with Abisil. However, the expression of some MAPKs (MAP4K4, MAP3K7, MAPK9, ZAK, MAPK1) was upregulated (or was detected only in non-treated cells).

## DISCUSSION

Terpenoids are undeservedly neglected in the study of geroprotective properties, except for carotenoids only [31]. At the same time, it is well documented that limonene [32], dibornol [33], betulinic [34], ursolic, and oleanolic acids [35] have some potential properties for preventing or alleviating diseases associated with aging.

Previously it was revealed that natural conifer terpenoids demonstrate anti-proliferative, pro-apoptotic activity and have anti-angiogenic potential. [17]. Earlier, we also investigated *in vitro* anti-aging and anti-cancer properties of Abisil, a complex mixture of diterpene and triterpene acids and esters with cyclic and acyclic monoterpenes from natural extract of Siberian fir [16]. We have shown that it restores the expression level of some prolongevity genes in old cells, including those involved in MAPK-, FOXO- and HIF-1 signaling pathways. In the present work we focused on the assessment of metabolic and cellular effects of Abisil, including mitochondrial function, autophagy whole transcriptome and proteome profiling.

Mitochondrial function relies on mitochondrial quality control and homeostasis. Mitohormesis is a process in which low, non-cytotoxic concentrations of reactive oxygen species promote mitochondrial homeostasis [36]. At a hormesis a dose response has either J-shape or an inverted U-shape dependency [37]. Exposure to low doses leads to protective and favorable reactions, while exposure to high doses is disruptive and harmful. [38]. There are several observations that confirm stimulation effects of Abisil at small doses. As shown in the present paper, while in low concentration Abisil had an antioxidant effect and at a higher concentration it had a prooxidant action. In addition, treatment of embryonic pulmonary fibroblast cell lines with Abisil revealed a dose-dependent metabolic activation, evaluated using the MTS test.

Sometimes hormetins have genotoxic effects, especially at high doses. There are complex transcriptomic markers of genotoxicity, e.g. combination of increased expression levels of *ATF3*, *CDKN1A* and *GADD45A* genes [39]. In the case of Abisil we could not observe any significant increase (2 times and more, FDR<0.05) in activity of these genes neither at low nor at high concentrations of the drug (Supplementary Tables 1, 2). At the same time, we noted statistically significant changes (both increase and decrease) in differential expression of genes, involved in the major DNA repair pathways: nucleotide excision repair, mismatch repair and homologous recombination.

Most likely Abisil introduces a shift in cell energy metabolism: whereas overall oxygen consumption rate was intact, the activity of glycolysis was significantly decreased. At the same time, we observed a decrease in mitochondrial potential, reduce in ROS levels and found a predominant decrease in the expression of genes involved in oxidative phosphorylation (namely those that are encoded in mitochondrial genome). Moreover, the effect of Abisil treatment extends not only to energy metabolism, but also causes a significant transcriptional reprogramming spanning thousands of genes involved in key cell pathways: we noted that about 15–20% of genes decrease their expression both at the transcriptome and proteome levels.

Treatment of cells with Abisil at a lowest concentration also resulted in bidirectional changes in the expression of ribosomal proteins and stimulation of amino acid biogenesis genes transcription. Impaired ribosome biogenesis is associated with cancer and aging [40]. At the proteome level, Abisil drops the expression of thousands of genes and downregulated proteins involved in the initiation of transcription and translation.

Abisil may contribute to the activation of VEGF pathway (Supplementary Figure 1), which regulates vascular development and blood and lymphatic vessel function [41]. It also activated T- and B-cell pathways, which can potentially delay immunosenescence.

In the present study, we demonstrated that Abisil (in a concentration of 50 μg/ml) effectively stimulated autophagy and reduced mitochondrial potential but did not decrease the number of mitochondrial DNA. It also induced drop of mitochondrial potential, without induction of mitophagy. Abisil did not cause mitophagy in MRC5-SV40 and LECH-4 cells. At the same time, it decreased the transcription levels of genes encoding subunits of mitochondrial complexes I, III, IV, V and the content of cytochrome c protein. It could be a mechanism for observed antioxidant effect and makes Abisil similar to metformin, that can inhibit complex I in intact cells [42]. In addition, it was recently observed that the mild depolarization of the inner mitochondrial membrane is another cause of the reduction of mitochondrial ROS [43].

As outer membrane of mitochondria is non-specifically permeable to all low-molecular-weight solutes, whereas the inner membrane is impermeable except through specific transporters [44], effects observed in this study could be explained by integration of one of the Abisil components into the outer membrane of mitochondria. That could lead to the decrease of mitochondrial potential without severe changes in oxygen consumption rate. ROS level reduction should be connected partially to increase of NADH oxidoreductases (Supplementary Figure 2) and superoxide dismutase 2. In addition, polyprenols, which are about 1.5% of Abisil, can act as scavengers of ROS [31].

In our opinion, it may be quite difficult to identify the main mechanism of action of Abisil, revealing its direct targets. There are at least two reasons for this. First, it is a multicomponent substance of natural origin, which is likely to have a complex impact on a cell. Secondly, at the transcriptomic and proteomic levels, we observe not only the primary effect (activation/inhibition of direct targets), but also all downstream events, such as activation of autophagy, cell cycle suppression, and so on. Moreover, downstream events on their scale, apparently, significantly exceed the primary effect, because transcriptomic and proteomic changes caused by Abisil treatment affect thousands of genes.

Regarding other transcriptomic and proteomic changes, Abisil led to a decrease in the expression of genes involved in the cell cycle and related processes. It is consistent with the results of the studies demonstrating that natural terpenoids may induce cell cycle arrest [17, 45]. Moreover, terpenoids are considered as potential chemopreventive and anti-cancer agents for tumor therapy [45, 46].

Since Abisil reduced the activity of fatty acid metabolism genes, it is expedient to test its effect on a metabolic syndrome and obesity. At least, switching from glucose metabolism to fatty acid metabolism, for example, with aging, increases the risk of ischemic myocardial damage due to free radicals and damage to DNA: beta-oxidation of fatty acids involves more oxygen than oxidation of pyruvate, and therefore provokes generation of more ROS [47]. PPARδ is a fatty acid sensor that enhances mitochondrial oxidation. Abisil significantly activates PPARδ expression both at low and high concentrations. Agonists of PPARδ affect energy homeostasis, anti-inflammation and insulin sensitivity and present an attractive target in Alzheimer’s disease pathogenesis [48].

On a proteome level Abisil treatment resulted in downregulation of mTOR, RICTOR, and Ras-related protein Rab-13. It is known that attenuation of TORC1 signaling delays replicative and oncogenic RAS-induced senescence [49]. Abisil decreases the expression of TGFBR1 receptor, which is evidently involved in tissue fibrosis [50]. Fibrosis is associated with upregulation of RBL1 [51], CDK9 [52], Renin receptor [53], mitogen-activated protein kinase p38α (*Mapk14* gene) [54]. TWEAK signals through its receptor, fibroblast growth factor inducible 14 (Fn14; TNFRSF12A) also modulates fibrosis in several organs, including heart, kidney, colon, and muscle [55]. Remarkably, all these target genes are downregulated by Abisil in the cell proteome.

Induction of cytosolic DNA-sensing pathway and innate and adaptive immune response and a bidirectional impact on endocytosis by Abisil proposes its antimicrobial and anti-viral activity. Considering the ongoing Covid-19 pandemic, these effects may be useful in prevention and treatment.

In conclusion, in small doses, Abisil exerted an antioxidant and anabolic effects, stimulated vascular growth factors and adaptive immunity genes. The mechanism of its action can include mitohormesis, since it affected autophagy and mitophagy, as well as the respiratory function of mitochondria. Abisil has a potential as anti-fibrotic, anti-metabolic drug and immunomodulatory substance, affecting many targets and cellular pathways.

## Supplementary Material

Supplementary Figures

Supplementary Table 1

Supplementary Table 2

Supplementary Table 3

Supplementary Table 4

Supplementary Table 5

Supplementary Table 6

Supplementary Table 7

Supplementary Table 8

Supplementary Table 9

Supplementary Table 10
