# Supplementary material for: Effects of Siberian fir terpenes extract Abisil on antioxidant activity, autophagy, transcriptome and proteome of human fibroblasts
Source: Aging (Albany NY). 2021 Aug 24;13(16):20050–80. doi: 10.18632/aging.203448 (PMC8436938; doi:10.18632/aging.203448)
Supplement: Supplementary Figures [file aging-13-203448-s001.pdf]

SUPPLEMENTARY FIGURES

A

KEGG pathway name	genes in pathway	GSEA min p (up-reg)	GSEA min p (down-reg)
Carbon metabolism	78	0.04	0.004
2-Oxocarboxylic acid metabolism	14	0.2	0.003
Fatty acid metabolism	36	0.2	0.0002
Biosynthesis of amino acids	49	0.0006	8.0603E-05
Amino sugar and nucleotide sugar metabolism	30	0.04	0.03
Inositol phosphate metabolism	39	0.02	0.4
Oxidative phosphorylation	109	0.003	1.972E-06
Fatty acid elongation	21	0.5	0.0003
Biosynthesis of unsaturated fatty acids	18	0.3	0.0002
Pyrimidine metabolism	34	0.02	0.2
Glycine, serine and threonine metabolism	18	0.06	0.02
Cysteine and methionine metabolism	29	0.1	0.1
Valine, leucine and isoleucine degradation	32	0.1	0.1
Valine, leucine and isoleucine biosynthesis	2	1.0	1.0
Lysine degradation	43	0.04	0.05
Arginine biosynthesis	9	0.6	0.2
Arginine and proline metabolism	29	0.03	0.005
N-Glycan biosynthesis	33	0.5	0.02
Mucin type O-glycan biosynthesis	11	0.4	0.07
Other types of O-glycan biosynthesis	15	0.03	0.07
Glycosaminoglycan biosynthesis - chondroitin	13	0.002	0.006
Glycosaminoglycan biosynthesis - heparan	11	0.03	0.2
Folate biosynthesis	12	0.3	0.04

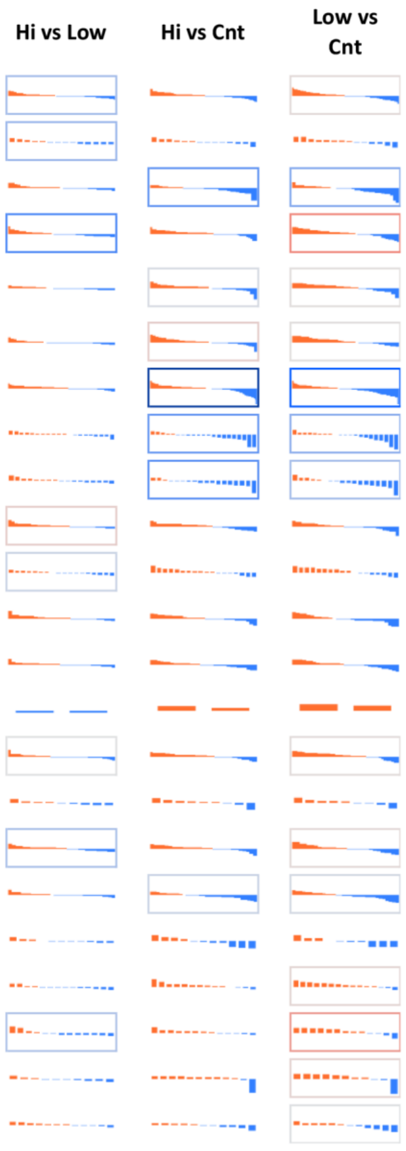

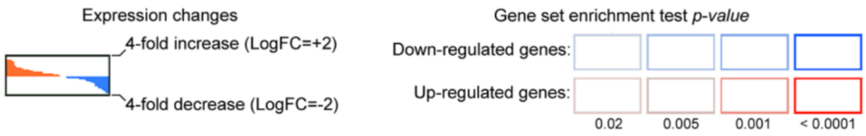

B

KEGG pathway name	genes in pathway	GSEA min p (up-reg)	GSEA min p (down-reg)
RNA polymerase	28	0.003	0.09
Basal transcription factors	30	0.002	0.0010
Spliceosome	110	1.4076E-05	8.6847E-05
Ribosome	130	9.8511E-11	0.06
Aminoacyl-tRNA biosynthesis	37	0.5	0.02
RNA transport	125	0.0005	3.9509E-07
mRNA surveillance pathway	66	0.001	0.02
Ribosome biogenesis in eukaryotes	64	0.1	0.006
Protein export	21	0.03	1.7607E-05
Protein processing in endoplasmic reticulum	122	0.03	0.004
SNARE interactions in vesicular transport	24	0.003	0.4
Ubiquitin mediated proteolysis	93	0.002	0.0008
Proteasome	39	0.10	0.002
RNA degradation	59	0.002	0.006
DNA replication	32	0.004	3.6776E-05
Base excision repair	28	0.10	0.01
Nucleotide excision repair	37	0.008	5.9647E-05
Mismatch repair	19	0.006	0.0002
Homologous recombination	28	0.001	0.009
Non-homologous end-joining	8	0.06	0.07
Fanconi anemia pathway	34	0.01	0.2
ABC transporters	11	0.3	0.06

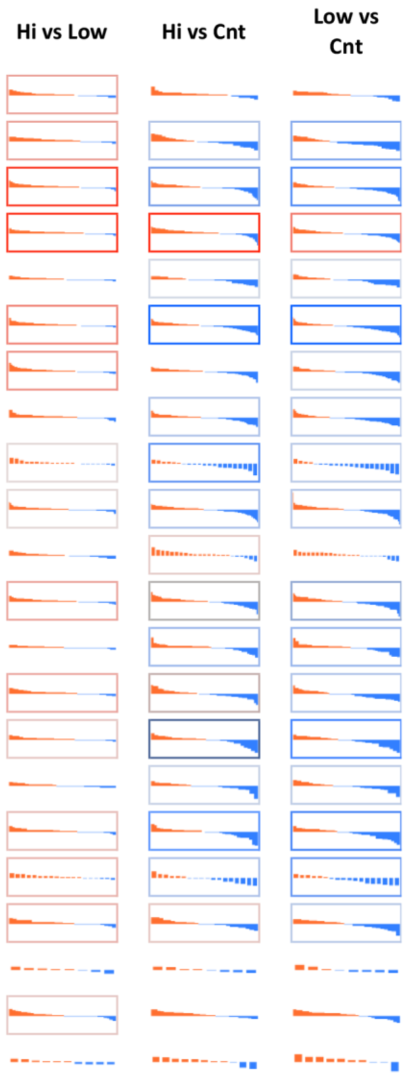

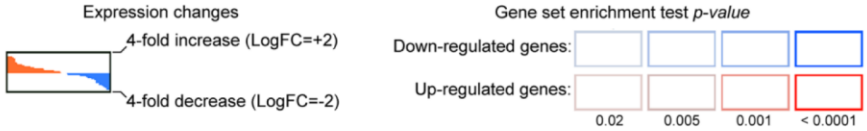

C

KEGG pathway name	genes in pathway	GSEA min p (up-reg)	GSEA min p (down-reg)	Hi vs Low	Hi vs Cnt	Low vs Cnt
Ras signaling pathway	91	0.08	0.2			
Rap1 signaling pathway	85	0.1	0.3			
MAPK signaling pathway	136	0.06	0.1			
ErbB signaling pathway	48	0.01	0.09			
Wnt signaling pathway	74	0.2	0.3			
Notch signaling pathway	29	0.04	0.03			
Hedgehog signaling pathway	22	0.2	0.5			
TGF-beta signaling pathway	43	0.3	0.09			
Hippo signaling pathway	81	0.007	0.03			
Hippo signaling pathway - multiple species	16	0.04	0.5			
VEGF signaling pathway	30	0.005	0.1			
JAK-STAT signaling pathway	53	0.2	0.8			
NF-kappa B signaling pathway	40	0.04	0.2			
TNF signaling pathway	55	0.007	0.04			
HIF-1 signaling pathway	59	0.1	0.01			
FoxO signaling pathway	69	0.009	0.5			
Calcium signaling pathway	56	0.6	0.2			
Phosphatidylinositol signaling system	53	0.0008	0.2			
Phospholipase D signaling pathway	64	0.3	0.1			
Sphingolipid signaling pathway	63	0.003	0.1			
cAMP signaling pathway	71	0.3	0.1			
cGMP-PKG signaling pathway	66	0.2	0.2			
PI3K-Akt signaling pathway	144	0.4	0.1			
AMPK signaling pathway	67	0.2	0.002			
mTOR signaling pathway	85	0.04	0.07			
Neuroactive ligand-receptor interaction	23	1.0	0.9			

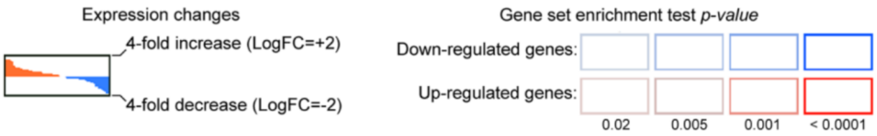

D

KEGG pathway name	genes in pathway	GSEA min p (up-reg)	GSEA min p (down-reg)
Cytokine-cytokine receptor interaction	35	0.8	0.9
ECM-receptor interaction	28	0.07	0.003
Cell adhesion molecules (CAMs)	35	0.3	0.8
Endocytosis	166	0.0009	7.4532E-05
Phagosome	65	0.2	0.5
Lysosome	85	8.9928E-05	0.008
Peroxisome	45	0.1	0.07
Autophagy - animal	82	0.1	0.02
Regulation of actin cytoskeleton	109	0.002	0.2
Cell cycle	97	0.0001	2.7889E-05
Oocyte meiosis	70	0.04	0.01
Apoptosis	81	0.008	0.1
Apoptosis - multiple species	18	0.06	0.6
p53 signaling pathway	44	0.01	0.1
Focal adhesion	100	0.02	0.0005
Adherens junction	49	9.9419E-05	0.007
Tight junction	85	0.2	0.08
Gap junction	40	0.10	0.3
ling pathways regulating pluripotency of stem cells	56	0.008	0.04
RIG-I-like receptor signaling pathway	34	0.04	0.2
Cytosolic DNA-sensing pathway	34	0.003	0.2
Natural killer cell mediated cytotoxicity	40	0.2	0.6
Antigen processing and presentation	30	0.8	0.02
T cell receptor signaling pathway	46	0.003	0.2
B cell receptor signaling pathway	34	0.001	0.08
Fc epsilon RI signaling pathway	28	0.08	0.05
Fc gamma R-mediated phagocytosis	51	0.01	0.2
Leukocyte transendothelial migration	47	0.006	0.3
Intestinal immune network for IgA production	4	0.9	1.0
Chemokine signaling pathway	68	0.08	0.1

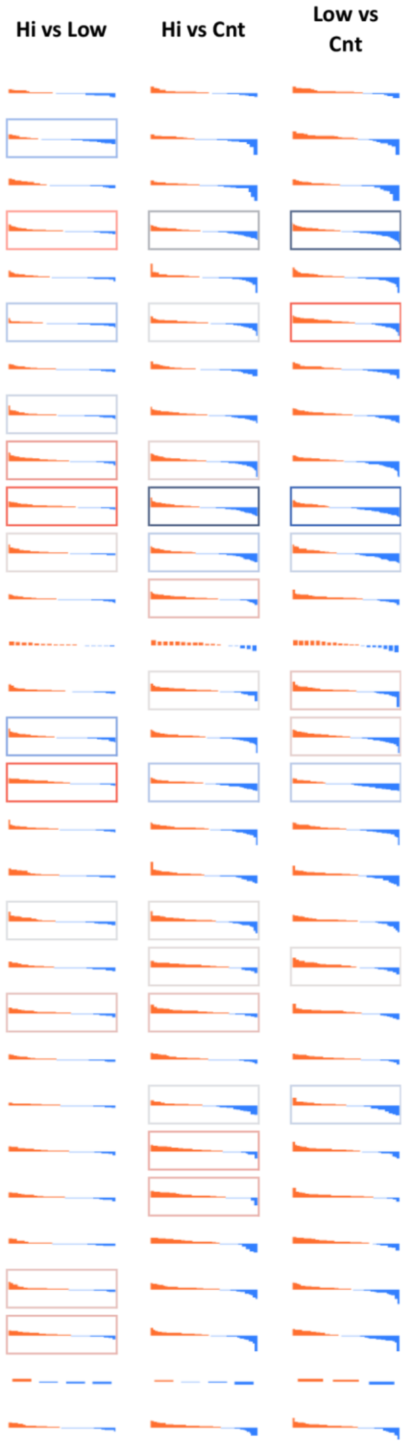

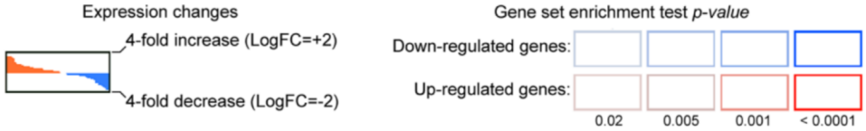

E

KEGG pathway name	genes in pathway	GSEA min p (up-reg)	GSEA min p (down-reg)
Insulin secretion	31	0.8	0.3
Insulin signaling pathway	77	0.02	0.2
Glucagon signaling pathway	51	0.2	0.05
Regulation of lipolysis in adipocytes	19	0.9	0.3
Adipocytokine signaling pathway	34	0.04	0.2
PPAR signaling pathway	23	0.01	0.09
GnRH signaling pathway	45	0.06	0.2
Melanogenesis	44	0.2	0.1
Renin secretion	21	0.9	0.2
Renin-angiotensin system	9	0.7	0.03
Aldosterone synthesis and secretion	36	0.6	0.02
Cardiac muscle contraction	35	0.06	0.009
Adrenergic signaling in cardiomyocytes	59	0.2	0.007
Vascular smooth muscle contraction	42	0.02	0.5
Protein digestion and absorption	28	0.2	0.03
Fat digestion and absorption	8	0.2	0.4
Vitamin digestion and absorption	6	0.3	0.5
Mineral absorption	19	0.1	0.0007

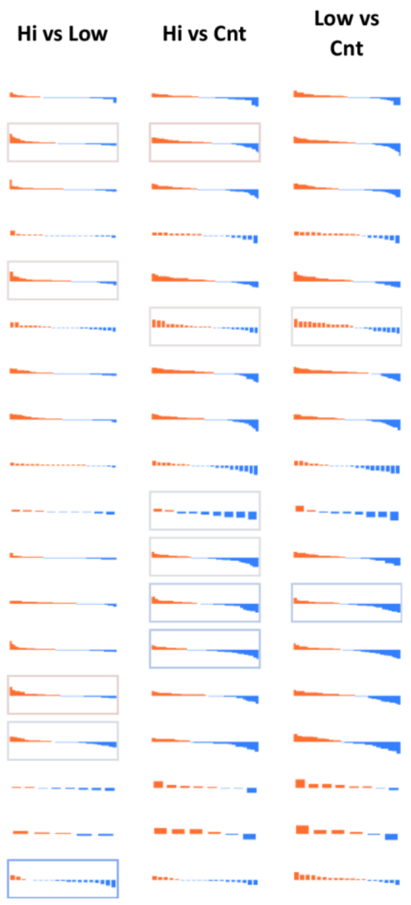

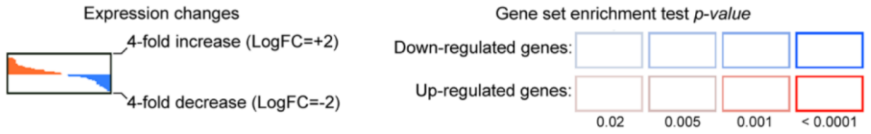

Supplementary Figure 1. Differential expression profiles of genes participating KEGG pathways according to the RNA-Seq data for MRC5-SV40 cell line treated with Abisil. On the figure, each cell demonstrates the sorted expression level log fold changes after Abisil treatment (red – upregulation, blue – downregulation) for genes participating a current KEGG pathway (vertical axis range is from 4-fold downregulation to 4-fold upregulation). Cell borders indicate whether a pathway is enriched with up- (red border) or downregulated (blue border) genes. *GSEA min p (up/down-reg)* – minimal p-value in a gene set enrichment analyses (GSEA; Fisher's exact test) for the pathway. *Hi vs Low* – comparison of cells treated with 50 and 5 µg/ml; *Hi vs Cnt* – comparison of cells treated with Abisil 50 µg/ml and non-treated cells; *Low vs Cnt* – comparison of cells treated with Abisil 5 µg/ml and non-treated cells. (**A**) Cell metabolism. (**B**) Main cellular processes. (**C**) Cell signaling pathways. (**D**) Immune response pathways, cell cycle, apoptosis, autophagy. (**E**) Miscellaneous pathways.

A Abisil 5 µg/ml compared to non-treated cells

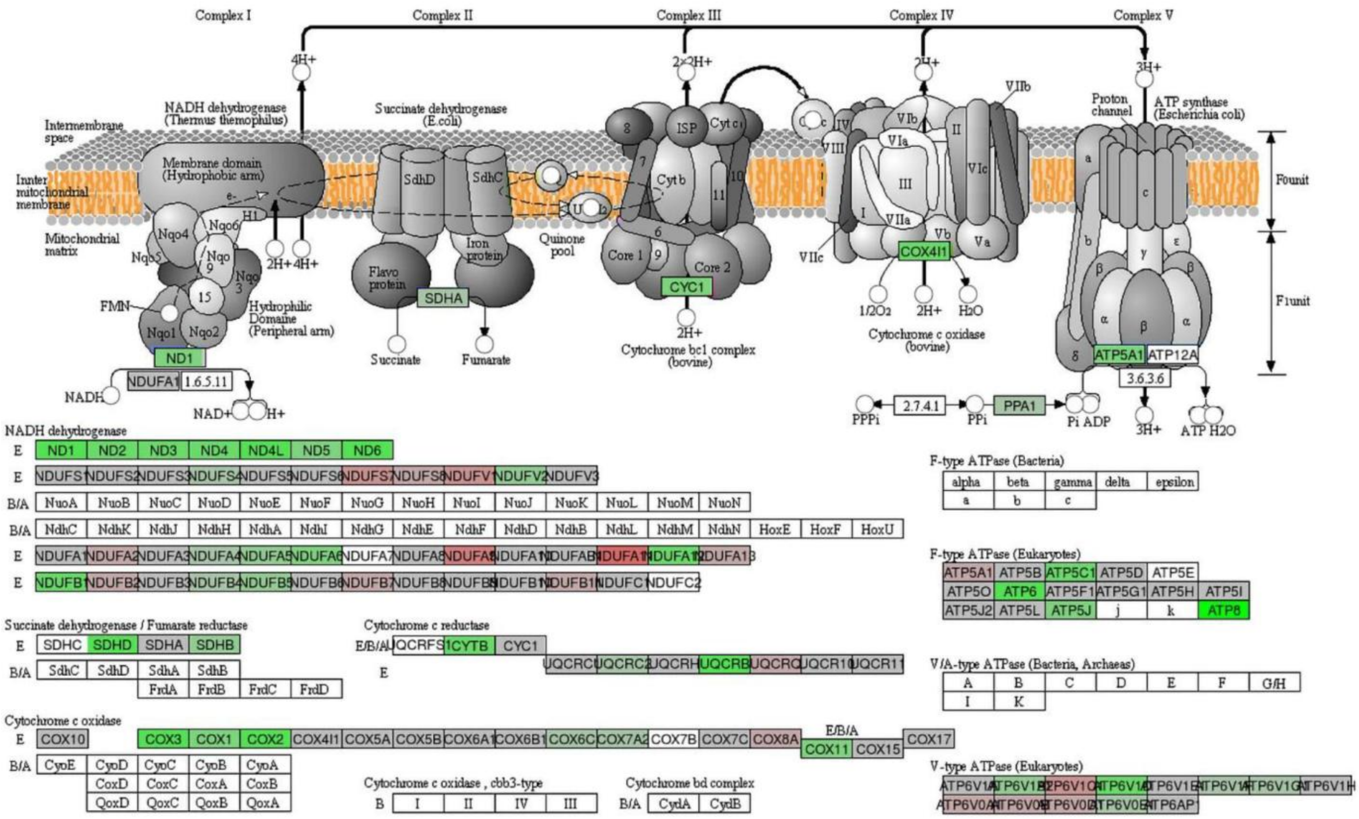

B Abisil 50 µg/ml compared to non-treated cells

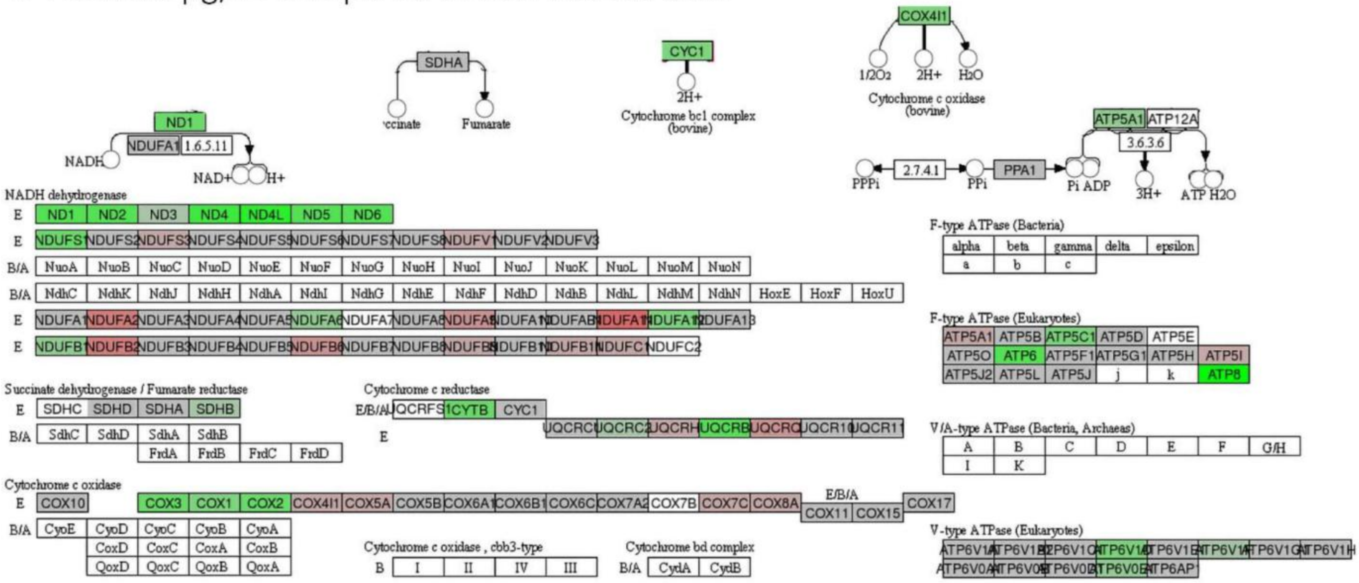

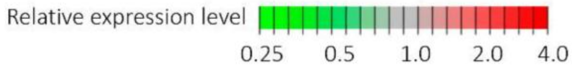

Supplementary Figure 2. Diagram illustrating expression changes of genes participating oxidative phosphorylation pathway (KEGG) introduced by Abisil treatment (MRC5-SV40 cell line): 5 µg/ml (**A**) and 50 µg/ml (**B**). Green – downregulation, red – upregulation.

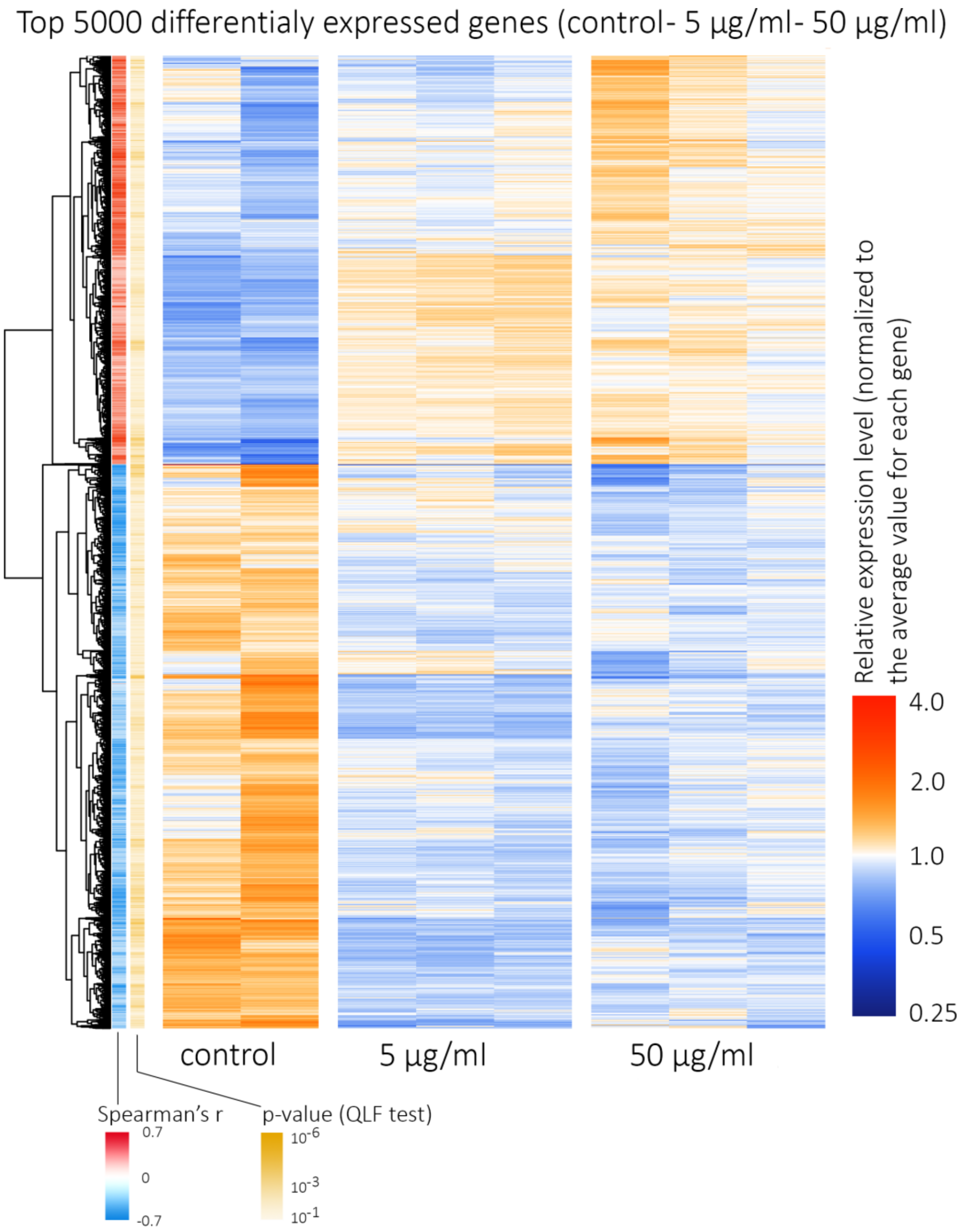

Supplementary Figure 3. Heatmap demonstrating expression level profiles of top 5000 genes (MRC5-SV40 cell line), expression of which gradually increases/decreases with the increase of Abisyl concentration (0 µg/ml — 5 µg/ml — 50 µg/ml).

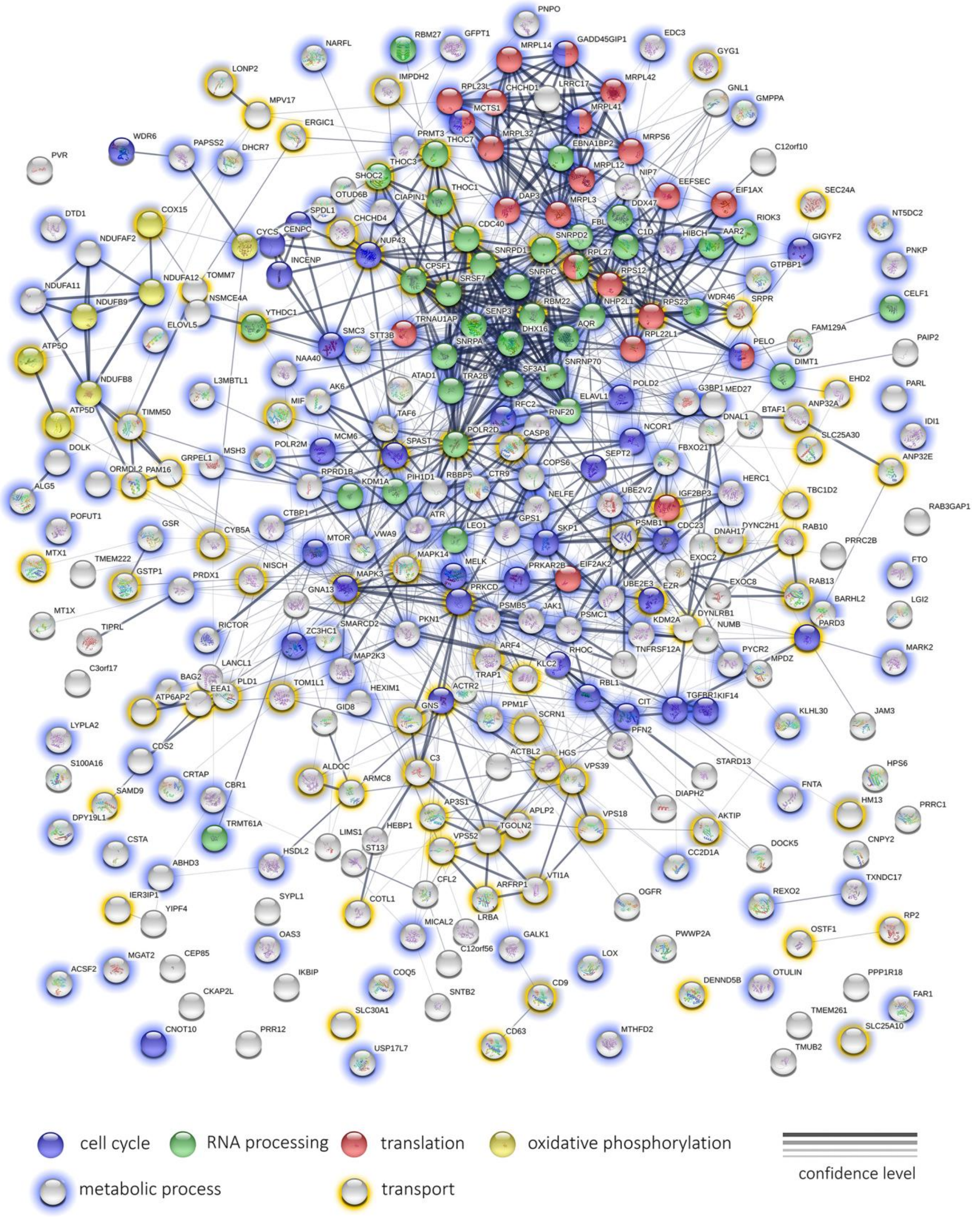

Supplementary Figure 4. The interaction network of top 300 proteins which content was decreased (or not detected) in MRC5-SV40 cells after Abisil treatment (50 µg/ml). The width of the connecting lines indicates the confidence level of protein interaction (best – mentions in curated databases, experimental data for human; worst – found interacting putative homologs in other organisms).

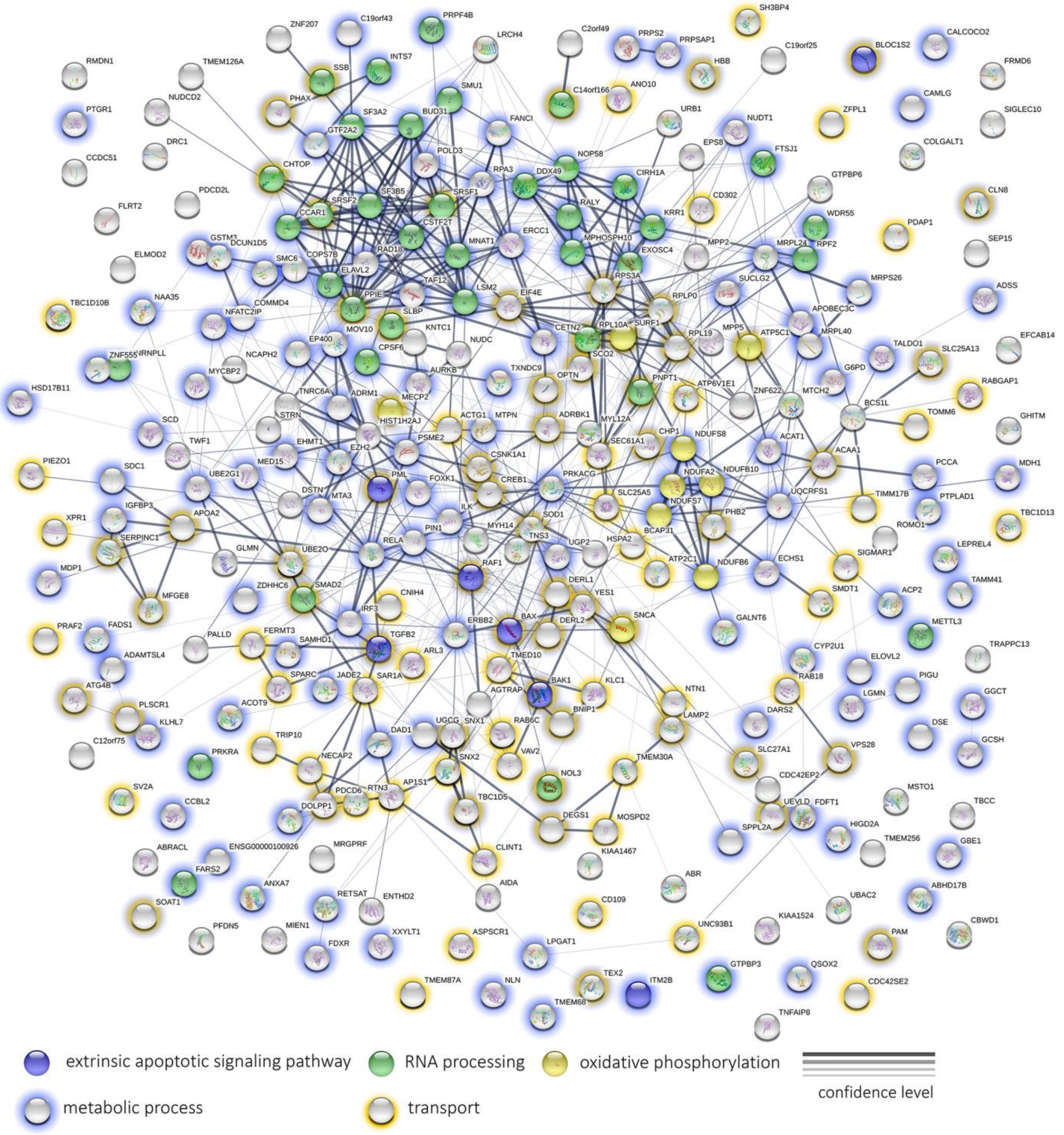

Supplementary Figure 5. The interaction network of top 300 proteins which content was increased in MRC5-SV40 cells after Abisil treatment (or detected only in treated cells). The width of the connecting lines indicates the confidence level of protein interaction (best – mentions in curated databases, experimental data for human; worst – found interacting putative homologs in other organisms).
